# Supplementary material for: Population-based socio-demographic household assessment of livelihoods and health among communities in Migori County, Kenya over multiple timepoints (2021, 2024, 2027): A study protocol
Source: PLoS One. 2021 Aug 25;16(8):e0256555. doi: 10.1371/journal.pone.0256555 (PMC8386871; doi:10.1371/journal.pone.0256555)
Supplement: S1 Appendix — Complete survey as it will be administered, although survey has been digitized into a tablet-based program. (DOCX) [file pone.0256555.s001.docx]

**COVID Screen**

*Please answer the questions below regarding recent symptoms.*

**cs_q1** Have you experienced a fever of 100.4F (38C) or greater, a new cough, new loss of taste or smell, or a shortness of breath within the past 14 days?

1. No
2. Yes 🡪 **STOP SURVEY**

**cs_q2** In the past 14 days, have you been tested positive for COVID-19?

1. No
2. Yes 🡪 **STOP SURVEY**

**cs_q3** To the best of your knowledge, in the past 14 days, have you been in close contact (within 6 feet (1.5 meter) or less for at least 10 minutes) with anyone while they had COVID-19?

1. No
2. Yes 🡪 **STOP SURVEY**

**[Only if the answer to all three questions is ‘No’ should you continue with the survey]**

**Interviewer Record**

**staff_id** Name of Interviewer: _____________________________________________________

**int_start** Interview date and time: __________________________________________________

**res_region** Household’s Region:

1. North Kamagambo

**res_area_nk** Household’s Area:

- - 1. Kameji
    2. North Kamwango
    3. South Kamwango

1. East Kamagambo

**res_area_ek** Household’s Area:

- - 1. East Kanyamamba
    2. Kagoro
    3. Kambija
    4. Kanyadieto
    5. Koluoch
    6. Kongoma
    7. Kongudi
    8. West Kanyamamba

1. Central Kamagambo

**res_area_ck** Household’s Area:

- - 1. Kabuoro
    2. Kamkuyu
    3. Kanyingombe
    4. Koderobara
    5. Township

1. South Kamagambo

**res_area_sk** Household’s Area:

1. Kamreri
2. Kanyawanga
3. Kanyimach
4. North Kanyajuok
5. South Kanyajuok
6. Central Kanyamkago 🡪 **res_area_uriri**
7. West Kanyamkago

**res_area_uriri** Household’s Area:

- - 1. Central Kawere Rateng
    2. East Kawere Rateng
    3. Kawere II
    4. Kojulu I B
    5. Kojulu I
    6. Lower Kojulu II
    7. Nyaobe
    8. Upper Kojulu II
    9. West Kawere Rateng

1. North Sakwa 🡪 **res_area_awendo**
2. Central Sakwa

**res_area_awendo** Household’s Area:

- - 1. Kakmasia East
    2. Kakmasia West
    3. Kamaure
    4. Kanyagwala
    5. Kasidula
    6. Rabondo

**res_hh** Is a male or female head of household home?

1. No 🡪 **STOP SURVEY**
2. Yes

**res_18** Confirm that the respondent is over the age of 18. [If the respondent is not over the age of 18, you are NOT allowed to proceed with the survey].

1. No 🡪 **STOP SURVEY**
2. Yes

**res_sex** What is the respondent's sex?

1. Woman

2. Man

**res_u5** Is there a child under 5 living in the household?

1. No
2. Yes

**res_longitude** Please write the longitude here:_____________________________

**res_latitude** Please write the latitude here:________________________________

**res_phone** What is the respondent’s phone number? _______________________

**Respondent Information**

**res_q1** What is your birthdate?____________________________

**[Record as DD/MM/YYY]**

**res_q2** How old were you at your last birthday?_______________________________

**[If year of birth and age do not align, reconfirm age and write best estimate]**

**Demographics**

**dem_q1** How long have you been living continuously in [Awendo, Rongo, Uriri]?

1. Always 🡪 **dem_q3**
2. 0-1 year 🡪 **dem_q2**
3. 1 year or longer 🡪 **dem_q1b**

**dem_q1b** Write number of years respondent has been living here if 1 or more years:________

**[Record in years]**

**dem_q2** Before living in [Awendo, Rongo, Uriri], did you previously live in:

1. Nairobi/Mombasa/Kisumu
2. Town
3. Countryside
4. Outside of Kenya

**dem_q3** What is your marital status?

1. Never married
2. Married monogamous [respondent's husband lives with no other women] 🡪 **dem_q3b**
3. Married polygamous [respondent's husband lives with her and at least one other woman]

**dem_q3a** How many co-wives live with you and your husband? _______🡪 **dem_q3b**

1. Cohabitating 🡪 **dem_q3b**
2. Separated
3. Divorced
4. Widowed

**dem_q3b** How many years have you been living with your current spouse/partner? ______________

**[Record 0 if less than 1 year]**

**dem_q4** What is your religion?

1. None
2. Catholic
3. Seventh-Day Adventist
4. Protestant
5. Roho Church
6. Legio Maria
7. African Independent Church
8. Hindu
9. Islam
10. OTHER: Specify: _____________________________

**dem_q5** What is the highest level of school that you completed?

1. No school
2. Class 1
3. Class 2
4. Class 3
5. Class 4
6. Class 5
7. Class 6
8. Class 7
9. Class 8
10. Form 1
11. Form 2
12. Form 3
13. Form 4
14. Some college
15. Completed college
16. Some university
17. Completed university
18. Post-graduate

**dem_q6** Have you ever contributed to the National Hospital Insurance Fund (NHIF)?

1. Active contributor
2. Inactive contributor
3. Never had NHIF
4. Don't know

**dem_q7** Do you and/or your partner have IDs?

1. Yes
2. No
3. Don't know

**dem_q9** Do any of your children have birth certificates?

1. I do not have children
2. Yes - they all do
3. No - none of them do
4. Some children have birth certificates while others do not

99. Don't know

**Household**

**hshld_q11** On a typical night, how many people eat in this household? In other words, how many people would you consider live in this household?______________

**[Including adults and children. Make sure to collect information about respondent him/herself, as well]**

**Please record information about each person living in the household. Make sure to include the respondent.**

| Name | Age  **[in years]** | Sex | Relationship to respondent | Highest Education  **[if age >= 3 years]** | Currently in School?  **[if age 3-24 years]** | School Type  **[if current school]** |
| --- | --- | --- | --- | --- | --- | --- |
|  |  | F or M |  |  | Y or N | Gov or Priv |
|  |  | F or M |  |  | Y or N | Gov or Priv |
|  |  | F or M |  |  | Y or N | Gov or Priv |
|  |  | F or M |  |  | Y or N | Gov or Priv |
|  |  | F or M |  |  | Y or N | Gov or Priv |
|  |  | F or M |  |  | Y or N | Gov or Priv |
|  |  | F or M |  |  | Y or N | Gov or Priv |
|  |  | F or M |  |  | Y or N | Gov or Priv |
|  |  | F or M |  |  | Y or N | Gov or Priv |
|  |  | F or M |  |  | Y or N | Gov or Priv |
|  |  | F or M |  |  | Y or N | Gov or Priv |
|  |  | F or M |  |  | Y or N | Gov or Priv |
|  |  | F or M |  |  | Y or N | Gov or Priv |
|  |  | F or M |  |  | Y or N | Gov or Priv |
|  |  | F or M |  |  | Y or N | Gov or Priv |
|  |  | F or M |  |  | Y or N | Gov or Priv |
|  |  | F or M |  |  | Y or N | Gov or Priv |
|  |  | F or M |  |  | Y or N | Gov or Priv |
|  |  | F or M |  |  | Y or N | Gov or Priv |
|  |  | F or M |  |  | Y or N | Gov or Priv |

**hshld_q1** Have you or a partner ever given birth?

1. No 🡪 **hshld_q18**
2. Yes

**hshld_q2** How many *total* pregnancies have you had that resulted in a *live* birth, including sons and daughters who were born but later died? ______________

**hshld_q3** How many of those sons and daughters are now living with you in your house? _____

**[Include those away at boarding school]**

**hshld_q4** How many sons and daughters are alive, but do not live with you? _____________

**[Exclude those away at boarding school]**

**hshld_q5** How many children have you or your partner given birth to who were born alive but later died?

1. None 🡪 **hshld_q8**
2. At least 1

**hshld_q5_atleast1** If "at least 1," how many children were born alive but later died? _________

**hshld_q6** How many boys have died? ___________

**hshld_q7** How many girls have died? ___________

**hshld_q8** How many pregnancies have you had that did not result in a live birth? ___________

**[That is, if the baby was not breathing or showing signs of life upon delivery]**

**hshld_q10** For each pregnancy that resulted in a live birth, how many children are 18 or younger, *including those children that were born alive and later died before their 19th birthday*? ___________

**[Please ensure that the respondent includes the number of children who were born alive and later died]**

**hshld_total_pregnancies_2013** In the last 5 years, how many *total* pregnancies have you had that resulted in a *live* birth, including sons and daughters who were born but later died? ___________

**hshld_q12** In the last 5 years, have any children in this household died before reaching their 5th birthday?

**[That is, children who were born in the last five years, and who died before they reached their *5th birthday*]**

1. No
2. Yes

**hshld_q13** How many of your children died before their fifth birthday in the last five years? __________

**[Include twins separately]**

**hshld_q15** Are there any children away at boarding school, or who do not stay in the household?

1. No 🡪 **hshld_q18**
2. Yes

**boys_hshldq15** How many boys stay outside the household? __________

**girls_hshldq15** How many girls stay outside the household? __________

**hshld_q18** How many total children have you or your partner given birth to? __________

**[includes children that were born alive but later died. Record 0 if none.]**

**Record information about all births in hshld_q18, whether still alive or not, starting with the first.**

| Name | Sex | Birthdate  **[MM/DD/YYY]** | Birth Certificate? | Alive? | Age at last birthday **[years]** | Death date  **[if deceased]** | Age at death  **[if deceased]** | Cause of death  **[if deceased]***** |
| --- | --- | --- | --- | --- | --- | --- | --- | --- |
|  | F or M |  | Y or N | Y or N |  |  |  |  |
|  | F or M |  | Y or N | Y or N |  |  |  |  |
|  | F or M |  | Y or N | Y or N |  |  |  |  |
|  | F or M |  | Y or N | Y or N |  |  |  |  |
|  | F or M |  | Y or N | Y or N |  |  |  |  |
|  | F or M |  | Y or N | Y or N |  |  |  |  |
|  | F or M |  | Y or N | Y or N |  |  |  |  |
|  | F or M |  | Y or N | Y or N |  |  |  |  |
|  | F or M |  | Y or N | Y or N |  |  |  |  |
|  | F or M |  | Y or N | Y or N |  |  |  |  |
|  | F or M |  | Y or N | Y or N |  |  |  |  |
|  | F or M |  | Y or N | Y or N |  |  |  |  |
|  | F or M |  | Y or N | Y or N |  |  |  |  |
|  | F or M |  | Y or N | Y or N |  |  |  |  |
|  | F or M |  | Y or N | Y or N |  |  |  |  |

***Causes of death

1. Anemia
2. Congenital Anomalies
3. Diarrhea
4. Injury
5. Malaria
6. Measles
7. Labor complication
8. Respiratory infection (not malaria)
9. Sickle Cell Disease
10. Other
11. Unknown

**[Record number in column]**

**hshld_q16** How many children were born to women that live in this household in the past year? This includes births to you or other women in the household. ___________

**[Questions hshld_q16a to hshld_q16a_5 to be answered for each woman]**

**hshld_q16a** How old was she [the FIRST woman who gave birth within the past year] when she had the baby?

1. Younger than 15 years old
2. Between 15 - 19 years old
3. Between 20 - 24 years old
4. Between 25 - 29 years old
5. Between 30 - 34 years old
6. Between 35 - 39 years old
7. Between 39 - 44 years old
8. Between 45 - 49 years old
9. Older than 49 years old

**hshld_q16a_2** How old was she [the SECOND woman who gave birth within the past year] when she had the baby?

1. Younger than 15 years old
2. Between 15 - 19 years old
3. Between 20 - 24 years old
4. Between 25 - 29 years old
5. Between 30 - 34 years old
6. Between 35 - 39 years old
7. Between 39 - 44 years old
8. Between 45 - 49 years old
9. Older than 49 years old

**hshld_q16a_3** How old was she [the THIRD woman who gave birth within the past year] when she had the baby?

1. Younger than 15 years old
2. Between 15 - 19 years old
3. Between 20 - 24 years old
4. Between 25 - 29 years old
5. Between 30 - 34 years old
6. Between 35 - 39 years old
7. Between 39 - 44 years old
8. Between 45 - 49 years old
9. Older than 49 years old

**hshld_q16a_4** How old was she [the FOURTH woman who gave birth within the past year] when she had the baby?

1. Younger than 15 years old
2. Between 15 - 19 years old
3. Between 20 - 24 years old
4. Between 25 - 29 years old
5. Between 30 - 34 years old
6. Between 35 - 39 years old
7. Between 39 - 44 years old
8. Between 45 - 49 years old
9. Older than 49 years old

**hshld_q16a_5** How old was she [the FIFTH woman who gave birth within the past year] when she had the baby?

1. Younger than 15 years old
2. Between 15 - 19 years old
3. Between 20 - 24 years old
4. Between 25 - 29 years old
5. Between 30 - 34 years old
6. Between 35 - 39 years old
7. Between 39 - 44 years old
8. Between 45 - 49 years old
9. Older than 49 years old

**hshld_q17** Are you or your partner currently pregnant?

1. No
2. Yes

**hshld_q17a** How many months pregnant are you or your partner? __________

**Economics**

*Now I am going to ask you about your family's business activities and finances. This is to help Lwala Community Alliance develop better programs for economic empowerment. All of this information will be kept completely confidential.*

**econ_q1** Who is the main bread winner for your household?

1. None
2. Female head of house
3. Male head of house
4. Co-wife
5. Parent(s)
6. Respondent's children
7. Co-wife's children
8. Aunt or Uncle
9. Grandparent
10. Friend

88. Other (specify)_________________

**econ_q2** Think about the main source of income for the household, what is the source of income? **[Read the list if necessary]**

1. Employed by government
2. Employed by private business
3. Employed by Lwala Community Alliance
4. Paid labour in private agriculture
5. Casual labor
6. Own agriculture
7. Own livestock breeding, animal products (cattle, goats, sheep)
8. Own small-scale animal (poultry, fish, rabbits)
9. Self-employed
10. Pensioner
11. Investments
12. Remittances

88. Other (specify) _____________________

99. Don't know

**econ_q3** Think about the secondary source of income for the household. What is the main secondary source of income?

1. Employed by government
2. Employed by private business
3. Employed by Lwala Community Alliance
4. Paid labour in private agriculture
5. Casual labor
6. Own agriculture
7. Own livestock breeding, animal products (cattle, goats, sheep)
8. Own small-scale animal (poultry, fish, rabbits)
9. Self-employed
10. Pensioner
11. Investments
12. Remittances

88. Other (specify) _____________________

99. Don't know

77. None

**econ_q4** On average, how much income does the household earn from all sources of income in one year?

1. Annual income: _____________________ (in KES)
2. Primarily in-kind payment or trades

99. Don’t Know

66. Prefer not to answer

**[Remind them to consider all sources of income, such as selling, remittances, farming income and help them to calculate the total]**

**econ_q5** How much does the household spend in a week on average?

1. Weekly expenditure: _____________________ (in KES)

99. Don’t Know

66. Prefer not to answer

**econ_q6** In the month when you make the MOST money, how much money do you make?

1. Monthly income: _____________________ (in KES)
2. Primarily in-kind payment or trades

99. Don’t Know

66. Prefer not to answer

**econ_q7** In the month when you make the LEAST money, how much money do you make?

1. Monthly income: _____________________ (in KES)
2. Primarily in-kind payment or trades

99. Don’t Know

66. Prefer not to answer

**econ_q8** Think about this time last year compared to now, do you feel that your household is better off, worse off or about the same, in terms of finances?

1. Better off
2. Worse off
3. About the same

99. Don’t know

**econ_q9** Do you own any of the following items in your household?

**[Read each item] [Select all that apply]**

1. Radio
2. Television
3. Refrigerator
4. Iron (charcoal or electric)
5. Kitchen sink
6. Mobile telephone
7. Non-mobile telephone
8. Computer of laptop
9. Bicycle
10. Motorbike
11. Car
12. Land with title deed
13. Well for water (in the yard)
14. Poultry or rabbits
15. Livestock (cows, goats, pigs)
16. Electricity

**econ_q43** Does any member of this household own a:

**[Select all that apply]**

1. Watch
2. Bicycle
3. Motorcycle or motor scooter
4. Animal-drawn cart
5. Car or truck
6. Boat with a motor

**econ_q12** Do you or a member of the household have a bank savings account? I do not want to know the exact institution or group you are a part of, just the types of savings accounts.

**[Read the list] [Select all that apply].**

1. Bank (such as Cooperative, KCB, or Equity)
2. Mobile banking (such as Mpesa or Mshwari)
3. Group savings
4. No bank account 🡪 **econ_q14**
5. Don't know 🡪 **econ_q14**
6. Prefer not to answer 🡪 **econ_q14**

**econ_q13** How much money do you have saved in all accounts?

1. Less than 1000 (KES)
2. 1001 – 10000
3. 10001 – 25000
4. 25001 – 50000
5. 50001 – 100000
6. 100001 – 300000
7. 300001 – 500000
8. More than 500000
9. Don't know
10. Prefer not to answer

**econ_q14** Do you regularly participate in a table banking group?

**[Explain table banking if unclear]**

1. Yes
2. No

99. Don’t know

66. Prefer not to answer

**econ_q15** Do you have any loans from the following institutions? I do not want to know any information about what specific institutions you might have loans from, just the types of institutions.

**[Read the list] [Select all that apply]**

1. No current loans 🡪 **econ_q19**
2. Table banking
3. Group bank
4. Individual Person
5. Microfinance Institution

88. Other (specify) ___________________

1. Don't know 🡪 **econ_q19**
2. Prefer not to answer 🡪 **econ_q19**

**econ_q16** What is the highest interest rate on your current loans?

1. Write percentage: _____________________ (in KES)

99. Don’t Know

66. Prefer not to answer

**econ_q18** What is your main reason for borrowing money? **[Select all that apply]**

1. To feed and clothe my family
2. School fees
3. Illness/Medical Costs
4. Small Business
5. Farming or livestock
6. Build/improve my house
7. Funeral
8. Wedding or other ceremony

88. Other (specify) ___________________

99. Don't know

**econ_q19** If you received additional income, what are TWO priorities you would spend money on? **[SELECT ONLY 2 PRIORITIES FROM THE LIST] [Read the list]**

1. Food
2. Clothes
3. Repair/expand house
4. Health services
5. School fees
6. Water or water-related equipment (tank, gutters)
7. Electricity supply
8. Buy car or motorbike
9. Open or expand a shop or business
10. Start professional training
11. Hire farm staff
12. Buy livestock goods/equipment
13. Buy agricultural goods/equipment
14. Rent or buy farm land
15. Greenhouse
16. Add to savings account
17. Other (specify) _________________________
18. Don't know

**econ_q20** Do you currently have an active kitchen garden?

1. No 🡪 **econ_q23**
2. Yes

**econ_q21** I am going to list some common crops grown in a kitchen garden. Please tell us if you have grown the following crops in the past year **[Select all that apply]**

1. Traditional vegetables (such as apotheosis, boo, den, osuga)
2. Exotic vegetables (such as kales, sakura)
3. Nuts/Njungu or beans
4. High value crops (such as ho ho, kiting, beet root, karat, Dania, morninga, roselle)
5. Don't know

**econ_q22** Do you grow crops from your kitchen garden for food only, food and income, or for income only?

1. Food only
2. Food and income
3. Income only
4. Don't know

**econ_q24** How many meals do you serve from your kitchen garden in a week?

1. Write number of meals: _____________________

99. Don’t Know

**econ_q25** Do you practice irrigation in your kitchen garden at any time of the year?

1. No 🡪 **econ_q23**
2. Yes

**econ_q26** Which kind of irrigation do you use in your kitchen garden at any time of the year?

**[Read all options] [Select all that apply]**

1. Drip
2. Water can
3. Foot pump
4. Mechanized/engine pump
5. Other (specify) ________________________

**econ_q23** How much money have you spent on food in the last week? ____________________

**[Give answer in KES]**

**econ_q27** Do you or any member of your household own or rent agricultural land that you regularly farm?

1. Yes – Owned
2. Yes - Owned and rented
3. Yes - Rent only
4. No 🡪 **econ_q32**

**econ_q28** What is the size of the agricultural land your household owns/rents, in square acres?

1. Write size in square acres: _____________________

99. Don’t Know

**econ_q29** Which crops do you grow on your own farm land? **[Do not prompt] [Select all that apply]**

1. Maize
2. Sugar cane
3. Cassava
4. Sweet potato
5. Kales
6. Beans
7. Fruit trees (such as avocados, papayas, or bananas)
8. Trees (non-food)
9. Other (specify) ____________________

**econ_q30** Do you practice irrigation on your farm at any time of the year?

1. No 🡪 **econ_q32**
2. Yes

**econ_q31** Which type of irrigation do you use on your farm?

1. Drip
2. Water can
3. Foot pump
4. Mechanized/engine pump
5. Other (specify) ________________________

**econ_q32** Have you or any member of your household received agricultural training?

1. Yes
2. No 🡪 **econ_q34**

99. Don’t know 🡪 **econ_q34**

**econ_q33** From which organization did you receive agriculture training?

1. Ministry of Agriculture
2. Other Government Agency
3. Dig (through Lwala)
4. Farmers United
5. Other non-governmental organization
6. Other (specify)_______________________
7. Don't know

**econ_q34** What is the highest educational level that the female household head/spouse reached?

1. Pre-primary, none, or other
2. Primary
3. Secondary or post-primary, vocational
4. College level or higher
5. There is no female household head/spouse

**econ_q35** What is the highest educational level that any member of the household reached?

1. Pre-primary, none, or other
2. Primary
3. Secondary or post-primary, vocational
4. College level or higher

**econ_q36** Over the past 7 days, did the household either purchase/consume/acquire any bread?

1. No
2. Yes

**econ_q37** Over the past 7 days, did the household either purchase/consume/acquire any meat or fish?

1. No
2. Yes

**econ_q38** Over the past 7 days, did the household either purchase/consume/acquire any ripe bananas?

1. No
2. Yes

**econ_q39** Does your household own any towels?

1. No
2. Yes

**econ_q40** Does your household own any thermos flasks?

1. No
2. Yes

**econ_q41** What is the predominant wall material of the main dwelling unit?

1. Finished walls (cement, stone with lime/cement, bricks, cement blocks, covered adobe, or wood planks/shingles)
2. Uncovered adobe, plywood, cardboard, reused wood, or corrugated iron sheets
3. Natural walls (cane/palm/trunks, grass/reeds, or mud/cow dung), no walls, bamboo with mud, stone with mud, or other

**econ_q42** What is the predominant floor material of the main dwelling unit?

1. Natural floor (earth/sand or dung) or palm/bamboo
2. Other (including wood planks/shingles, parquet or polished wood, vinyl or asphalt strips, ceramic tiles, cement, or carpet)

**Child Health**

**branch_u5count** How many children do you have that are less than 5 years old? ________

**Please fill in information for all children less than five years old.**

| **Name** | **Birthdate** | **Age** |
| --- | --- | --- |
|  |  |  |
|  |  |  |
|  |  |  |
|  |  |  |
|  |  |  |
|  |  |  |

**Please fill in the following information about the pregnancies and births for all children under 5 years of age.**

|  | Name_______ | Name_______ | Name_______ | Name_______ | Name_______ | Name_______ |
| --- | --- | --- | --- | --- | --- | --- |
| During you or your partner's pregnancy with this child, how many antenatal care (ANC) visits did you attend at a health facility before you delivered? | No.:________  99. Don’t know | No.:________  99. Don’t know | No.:________  99. Don’t know | No.:________  99. Don’t know | No.:________  99. Don’t know | No.:________  99. Don’t know |
| How many months pregnant were you when you first received antenatal care for this pregnancy? | Months:______ | Months:______ | Months:______ | Months:______ | Months:______ | Months:______ |
| Did your husband/partner attend any of the ANC visits with you? **[if female]** | 1. Yes  2. No  99. Don’t know | 1. Yes  2. No  99. Don’t know | 1. Yes  2. No  99. Don’t know | 1. Yes  2. No  99. Don’t know | 1. Yes  2. No  99. Don’t know | 1. Yes  2. No  99. Don’t know |
| Did you [husband/partner] ever attend any ANC visits with your partner?  **[if male]** | 1. Yes  2. No  99. Don’t know | 1. Yes  2. No  99. Don’t know | 1. Yes  2. No  99. Don’t know | 1. Yes  2. No  99. Don’t know | 1. Yes  2. No  99. Don’t know | 1. Yes  2. No  99. Don’t know |
| During this pregnancy, did the mother receive an injection in the arm to prevent the baby from getting tetanus, that is, convulsions after birth? | 1. Yes  2. No  99. Don’t know | 1. Yes  2. No  99. Don’t know | 1. Yes  2. No  99. Don’t know | 1. Yes  2. No  99. Don’t know | 1. Yes  2. No  99. Don’t know | 1. Yes  2. No  99. Don’t know |
| How many times did you or your partner receive such an injection? | 1. 1 time  2. 2 times  3. 3 or more  99. Don’t know | 1. 1 time  2. 2 times  3. 3 or more  99. Don’t know | 1. 1 time  2. 2 times  3. 3 or more  99. Don’t know | 1. 1 time  2. 2 times  3. 3 or more  99. Don’t know | 1. 1 time  2. 2 times  3. 3 or more  99. Don’t know | 1. 1 time  2. 2 times  3. 3 or more  99. Don’t know |
| Where did you or your partner give birth to this child?  **[see facility list; record number]** | No.__________  89. Your home  90. Other home  88. Other [**record below]** | No.__________  89. Your home  90. Other home  88. Other [**record below]** | No.__________  89. Your home  90. Other home  88. Other [**record below]** | No.__________  89. Your home  90. Other home  88. Other [**record below]** | No.__________  89. Your home  90. Other home  88. Other [**record below]** | No.__________  89. Your home  90. Other home  88. Other [**record below]** |
| Other: |  |  |  |  |  |  |
| Who assisted with the delivery of this child? | 1. No one  2. Clinician/nurse  3. Doctor  4. Midwife  5. Traditional birth attendant  6. Relative/friend  88. Other  **[record below]** | 1. No one  2. Clinician/nurse  3. Doctor  4. Midwife  5. Traditional birth attendant  6. Relative/friend  88. Other  **[record below]** | 1. No one  2. Clinician/nurse  3. Doctor  4. Midwife  5. Traditional birth attendant  6. Relative/friend  88. Other  **[record below]** | 1. No one  2. Clinician/nurse  3. Doctor  4. Midwife  5. Traditional birth attendant  6. Relative/friend  88. Other  **[record below]** | 1. No one  2. Clinician/nurse  3. Doctor  4. Midwife  5. Traditional birth attendant  6. Relative/friend  88. Other  **[record below]** | 1. No one  2. Clinician/nurse  3. Doctor  4. Midwife  5. Traditional birth attendant  6. Relative/friend  88. Other  **[record below]** |
| Other: |  |  |  |  |  |  |

**Now I would like to ask you some questions about cough and fever in your children under five years old.**

|  | Name_______ | Name_______ | Name_______ | Name_______ | Name_______ | Name_______ |
| --- | --- | --- | --- | --- | --- | --- |
| **R1.** In the last two weeks, has this child been ill with a fever at any time? | 1. Yes  2. No  99. Don’t know | 1. Yes  2. No  99. Don’t know | 1. Yes  2. No  99. Don’t know | 1. Yes  2. No  99. Don’t know | 1. Yes  2. No  99. Don’t know | 1. Yes  2. No  99. Don’t know |
| **R2.** In the last two weeks, has this child had an illness with a cough? | 1. Yes  2. No 🡪 **R4**  99. Don’t know 🡪 **R4** | 1. Yes  2. No 🡪 **R4**  99. Don’t know 🡪 **R4** | 1. Yes  2. No 🡪 **R4**  99. Don’t know 🡪 **R4** | 1. Yes  2. No 🡪 **R4**  99. Don’t know 🡪 **R4** | 1. Yes  2. No 🡪 **R4**  99. Don’t know 🡪 **R4** | 1. Yes  2. No 🡪 **R4**  99. Don’t know 🡪 **R4** |
| **R3.** Has this child had fast, short, rapid breaths or difficulty breathing any time in the last 2 weeks? | 1. Yes  2. No  99. Don’t know | 1. Yes  2. No  99. Don’t know | 1. Yes  2. No  99. Don’t know | 1. Yes  2. No  99. Don’t know | 1. Yes  2. No  99. Don’t know | 1. Yes  2. No  99. Don’t know |
| **R4.** | **[If R1 and R2 are No, exit table]** | **[If R1 and R2 are No, exit table]** | **[If R1 and R2 are No, exit table]** | **[If R1 and R2 are No, exit table]** | **[If R1 and R2 are No, exit table]** | **[If R1 and R2 are No, exit table]** |
| **R5.** Did you seek advice or treatment for this illness? | 1. Yes  2. No 🡪**skip to R10**  99. Don’t know🡪**skip to R10** | 1. Yes  2. No 🡪**skip to R10**  99. Don’t know🡪**skip to R10** | 1. Yes  2. No 🡪**skip to R10**  99. Don’t know🡪**skip to R10** | 1. Yes  2. No 🡪**skip to R10**  99. Don’t know🡪**skip to R10** | 1. Yes  2. No 🡪**skip to R10**  99. Don’t know🡪**skip to R10** | 1. Yes  2. No 🡪**skip to R10**  99. Don’t know🡪**skip to R10** |
| **R6.** Where did you seek advice or treatment?  **[see facility list; record number]** | No.__________  88. Other  **[Record below]** | No.__________  88. Other  **[Record below]** | No.__________  88. Other  **[Record below]** | No.__________  88. Other  **[Record below]** | No.__________  88. Other  **[Record below]** | No.__________  88. Other  **[Record below]** |
| **R7.** Other |  |  |  |  |  |  |
| **R8.** When you sought advice or treatment for the fever, was a malaria rapid diagnostic test (RDT) done? | 1. Yes  2. No  99. Don’t know | 1. Yes  2. No  99. Don’t know | 1. Yes  2. No  99. Don’t know | 1. Yes  2. No  99. Don’t know | 1. Yes  2. No  99. Don’t know | 1. Yes  2. No  99. Don’t know |
| **R9.** Did someone at the healthcare clinic diagnose your child with malaria? | 1. Yes  2. No  99. Don’t know | 1. Yes  2. No  99. Don’t know | 1. Yes  2. No  99. Don’t know | 1. Yes  2. No  99. Don’t know | 1. Yes  2. No  99. Don’t know | 1. Yes  2. No  99. Don’t know |
| **R10.** Did this child take any drugs for the illness? | 1. Yes  0. No **🡪 exit table** | 1. Yes  0. No **🡪 exit table** | 1. Yes  0. No **🡪 exit table** | 1. Yes  0. No **🡪 exit table** | 1. Yes  0. No **🡪 exit table** | 1. Yes  0. No **🡪 exit table** |
| **R11.** What treatment was given to this child?  **[Responses below]** | No.__________  99. Don’t know  88. Other [**record below]** | No.__________  99. Don’t know  88. Other [**record below]** | No.__________  99. Don’t know  88. Other [**record below]** | No.__________  99. Don’t know  88. Other [**record below]** | No.__________  99. Don’t know  88. Other [**record below]** | No.__________  99. Don’t know  88. Other [**record below]** |
| **R12.** Other: |  |  |  |  |  |  |
| **R13.** How long after the illness started did this child first take the medications? | 0. Same day  1. Next day  2. 2 days after  3. 3 days after  4. 4 days after  99. Don’t know | 0. Same day  1. Next day  2. 2 days after  3. 3 days after  4. 4 days after  99. Don’t know | 0. Same day  1. Next day  2. 2 days after  3. 3 days after  4. 4 days after  99. Don’t know | 0. Same day  1. Next day  2. 2 days after  3. 3 days after  4. 4 days after  99. Don’t know | 0. Same day  1. Next day  2. 2 days after  3. 3 days after  4. 4 days after  99. Don’t know | 0. Same day  1. Next day  2. 2 days after  3. 3 days after  4. 4 days after  99. Don’t know |

**Responses for R11 above:**

| 1 | SP/Fansidar |
| --- | --- |
| 2 | Chloroquine |
| 3 | Amodiaquine |
| 4 | Quinine |
| 5 | AL/Coartem |
| 6 | Artesunate |
| 7 | Other anti-malarial |
| 8 | Antibiotic pill/syrup |
| 9 | Antibiotic injection |
| 10 | Aspirin |
| 11 | Paracetamol |
| 12 | Ibuprofen |

**Now I would like to ask you questions about diarrhea in your children less than 5.**

|  | Name_______ | Name_______ | Name_______ | Name_______ | Name_______ | Name_______ |
| --- | --- | --- | --- | --- | --- | --- |
| In the last two weeks, has this child had diarrhea at any time? | 1. Yes  2. No **🡪 exit table**  99. Don’t know **🡪 exit table** | 1. Yes  2. No **🡪 exit table**  99. Don’t know **🡪 exit table** | 1. Yes  2. No **🡪 exit table**  99. Don’t know **🡪 exit table** | 1. Yes  2. No **🡪 exit table**  99. Don’t know **🡪 exit table** | 1. Yes  2. No **🡪 exit table**  99. Don’t know **🡪 exit table** | 1. Yes  2. No **🡪 exit table**  99. Don’t know **🡪 exit table** |
| Did you seek advice or treatment for the diarrhea? | 1. Yes  2. No **🡪 exit table**  99. Don’t know **🡪 exit table** | 1. Yes  2. No **🡪 exit table**  99. Don’t know **🡪 exit table** | 1. Yes  2. No **🡪 exit table**  99. Don’t know **🡪 exit table** | 1. Yes  2. No **🡪 exit table**  99. Don’t know **🡪 exit table** | 1. Yes  2. No **🡪 exit table**  99. Don’t know **🡪 exit table** | 1. Yes  2. No **🡪 exit table**  99. Don’t know **🡪 exit table** |
| Where did you seek advice or treatment?  **[see facility list; record number]** | No.__________  88. Other  **[Record below]** | No.__________  88. Other  **[Record below]** | No.__________  88. Other  **[Record below]** | No.__________  88. Other  **[Record below]** | No.__________  88. Other  **[Record below]** | No.__________  88. Other  **[Record below]** |
| Other: |  |  |  |  |  |  |

**chh_q26** Can you tell me what symptoms indicate that a child needs to be taken to a health facility? Please tell me all the symptoms that you can. **[Select all that apply]**

1. No answer given
2. Looks unwell or not playing normally
3. Fever or chills
4. Not eating or drinking
5. Vomits everything
6. Lethargic or difficult to wake
7. Persistent cough
8. Fast or difficult breathing
9. Diarrhea
10. Bloody stool
11. Convulsions
12. Other (specify) ________________________

**chh_q27** In your understanding, tell me some of the ways that you can identify a child who is malnourished? **[Select all that apply]**

1. No answer given
2. Sunken eyes
3. Decreased body weight
4. Discolored hair
5. Small body size
6. Thin limbs
7. Extended belly
8. Fatigue or lethargy
9. Flabby cheeks
10. Pale skin
11. Other (specify) ________________________

**Family Planning**

**fp_q1** Have you or your spouse ever used any method of family planning, that is contraception, or done something to delay or avoid getting pregnant?

1. Yes
2. No 🡪 **fp_q6**

99. Don’t know 🡪 **fp_q6**

**fp_q2** Are you or your spouse currently doing something or using any method of family planning to delay or avoid getting pregnant?

1. Yes
2. No 🡪 **fp_q5**

99. Don’t know 🡪 **fp_q5**

**fp_q3** Which method(s) are you or your spouse currently using to avoid getting pregnant?

**[Do not prompt] [Select All that Apply]**

1. IUD / IUCD / COIL
2. Injectables (such as Depo)
3. Implants (such as Implanon or Jadelle)
4. Pill
5. Condom
6. Female Condom
7. Female Sterilization
8. Male Sterilization
9. Diaphragm
10. Foam / Jelly
11. Lactational Amen Method
12. Rhythm Method
13. Withdrawal
14. Other Modern Method
15. Other Traditional method
16. Don't know

**fp_q4** When was this method of family planning given to you/your spouse, most recently?

1. 0-6 months ago
2. 7-12 months ago
3. 1-2 years ag
4. 2-3 years ago
5. 3-4 years ago
6. More than 4 years ago

99. Don't know

**fp_q5** Where did you/spouse receive this method of family planning the last time you got it?

**[Select from Facility List]**

1. Number from facility list: ____________

88. Other: ___________________________

**fp_q6** Do you or your spouse currently use male condoms to avoid getting pregnant or prevent sexually transmitted infections?

1. No
2. Yes

**fp_q17** Do you or your spouse currently use female condoms to avoid getting pregnant or prevent sexually transmitted infections?

1. No
2. Yes

**fp_q7** If you were to suggest using a condom to your partner, would you feel afraid that he/she would reject you?

1. No
2. Yes

**fp_q8** In the last year, how often did you talk with your spouse about family planning or contraception? **[only if married; see dem_q3]**

1. Never
2. Once or twice
3. Three or more times

**fp_q9** Would you say you approve or disapprove of using a family planning method to avoid pregnancy?

1. Disapprove
2. Approve
3. No opinion

99. Don’t know

**fp_q10** How old were you when you or your partner gave birth to your first child? _________

**[Only if have had child before; see hshld_q1]**

**fp_q20** When did your last menstrual period start? **[Only if female; see res_sex]**

1. Knows approximate date

**date_fp_q20** First day of last menstruation/period:_________________

1. In menopause/Has had hysterectomy
2. Before last birth/Before current pregnancy
3. Never menstruated

**fp_q18** When you got pregnant, did you want to get pregnant at that time? **[only if currently pregnant; see hshld_17]**

1. No
2. Yes 🡪 **skip to table below**

**fp_q19** Did you want to have a baby later on or did you not want any (more) children?

1. Later
2. No more

**Please fill in the following information for each child under 5 years old.**

|  | Name_______ | Name_______ | Name_______ | Name_______ | Name_______ | Name_______ |
| --- | --- | --- | --- | --- | --- | --- |
| **R1.** When you got pregnant with (NAME), did you want to get pregnant at that time? | 1. Yes**🡪 R5**  0. No | 1. Yes**🡪 R5**  0. No | 1. Yes**🡪 R5**  0. No | 1. Yes**🡪 R5**  0. No | 1. Yes**🡪 R5**  0. No | 1. Yes**🡪 R5**  0. No |
| **R2.** Did you want to have a baby later on, or did you not want any (more) children? | 1. Wait longer  2. No more children 🡪 **R5**  88. Other 🡪 **R5**  **[record below]** | 1. Wait longer  2. No more children 🡪 **R5**  88. Other 🡪 **R5**  **[record below]** | 1. Wait longer  2. No more children 🡪 **R5**  88. Other 🡪 **R5**  **[record below]** | 1. Wait longer  2. No more children 🡪 **R5**  88. Other 🡪 **R5**  **[record below]** | 1. Wait longer  2. No more children 🡪 **R5**  88. Other 🡪 **R5**  **[record below]** | 1. Wait longer  2. No more children 🡪 **R5**  88. Other 🡪 **R5**  **[record below]** |
| **R3.** Other: |  |  |  |  |  |  |
| **R4.** How much longer did you want to wait? | Months:_____  99. Don’t know | Months:_____  99. Don’t know | Months:_____  99. Don’t know | Months:_____  99. Don’t know | Months:_____  99. Don’t know | Months:_____  99. Don’t know |
| **R5.** Were you able to access contraception or family planning at the time that you conceived this baby? | 1. Yes  0. No | 1. Yes  0. No | 1. Yes  0. No | 1. Yes  0. No | 1. Yes  0. No | 1. Yes  0. No |

**fp_q21** Would you like to have (a/another) child, or would you prefer not to have any (more) children?

1. Have another child
2. No more/None 🡪 **fp_q16**
3. Cannot get pregnant 🡪 **fp_q16**
4. Undecided/Don't know 🡪 **fp_q16**

**fp_q15** How long would you like to wait from now before the birth of a/another child?

1. Months/years

**fp_q15_months** How many months? ___________________

1. Soon/Now 🡪 **skip to next survey section**
2. Respondent or partner cannot get pregnant
3. After marriage

88. Other (specify): __________________

99. Don't know

**fp_q16** Can you tell me why you are not using a method to avoid pregnancy?

1. Currently using contraception **[select if fp_q2 = 1]**

66. Currently pregnant **[select if hshld_17 = 1]**

1. Not married
2. Not having sex
3. Infrequent sex
4. Menopausal/hysterectomy
5. Can't get pregnant
6. Not menstruated since last birth
7. Breastfeeding
8. Up to god/fatalistic
9. Respondent opposed
10. Partner opposed
11. Others opposed
12. Religious prohibition
13. Knows no method
14. Knows no source
15. Side effects/health concerns
16. Lack of access/too far
17. Costs too much
18. Preferred method not available
19. No method available
20. Inconvenient to use
21. Interferes with body's normal processes
22. Other __________________________
23. Don't know

**Nutrition**

**nutr_q36** In the past 4 weeks, was there ever no food to eat of any kind in your house because of lack of resources to get food?

1. No 🡪 **nutr_q38**
2. Yes

**nutr_q37** How often did this happen in the past 4 weeks?

1. Rarely (1-2 times)
2. Sometimes (3-10 times)
3. Often (more than 10 times)

**nutr_q38** In the past 4 weeks, did you or any household member go to sleep at night hungry because there was not enough food?

1. No 🡪 **nutr_q40**
2. Yes

**nutr_q39** How often did this happen in the past 4 weeks?

1. Rarely (1-2 times)
2. Sometimes (3-10 times)
3. Often (more than 10 times)

**nutr_q40** In the past 4 weeks, did you or any household member go a whole day and night without eating anything at all because there was not enough food?

1. No 🡪 **nutr_q1**
2. Yes

**nutr_q41** How often did this happen in the past 4 weeks?

1. Rarely (1-2 times)
2. Sometimes (3-10 times)
3. Often (more than 10 times)

**nutr_q1** Have you or any of your children ever been referred to the facility for malnutrition?

1. No
2. Yes

**nutr_q1b** Date (DD/MM/YYY): _______________________

**nutr_infant** Do you have a child who is less than 24 months old?

1. No 🡪 **skip to table below**
2. Yes

**nutr_q2** Has the child ever been breastfed?

1. No 🡪 **skip to table below**
2. Yes

**Immediate_breastfeeding** How long after birth did you first put this child to the breast?

1. Immediately
2. Less than 24 hours after birth

**hours_imm_breastfeeding** Hours: _________________

1. More than 24 hours after birth

**days_imm_breastfeeding** Days: ___________________

**Now I would like to ask you questions about breastfeeding of your other young children. Please fill out this table for all children under five years of age.**

|  | Name_______ | Name_______ | Name_______ | Name_______ | Name_______ | Name_______ |
| --- | --- | --- | --- | --- | --- | --- |
| **R1.** Has this child ever been breastfed? | 1. Yes  0. No 🡪 **R3** | 1. Yes  0. No 🡪 **R3** | 1. Yes  0. No 🡪 **R3** | 1. Yes  0. No 🡪 **R3** | 1. Yes  0. No 🡪 **R3** | 1. Yes  0. No 🡪 **R3** |
| **R2.** Was this child breastfed yesterday during the day or at night? | 1. Yes 🡪 **R5**  0. No | 1. Yes 🡪 **R5**  0. No | 1. Yes 🡪 **R5**  0. No | 1. Yes 🡪 **R5**  0. No | 1. Yes 🡪 **R5**  0. No | 1. Yes 🡪 **R5**  0. No |
| **R3.** Sometimes babies are fed breast milk in different ways, for example by spoon, cup or bottle. This can happen when the mother cannot always be with her baby. Sometimes babies are breastfed by another woman, or given breast milk from another woman by spoon, cup or bottle or some other way. This can happen if a mother cannot breastfeed her own baby. | | | | | | |
| **R4.** Did this child consume breast milk in any of these ways yesterday during the day or at night? | 1. Yes  0. No | 1. Yes  0. No | 1. Yes  0. No | 1. Yes  0. No | 1. Yes  0. No | 1. Yes  0. No |
| **R5.** Was this child given any vitamin drops or other medicines as drops yesterday during the day or at night? | 1. Yes  0. No | 1. Yes  0. No | 1. Yes  0. No | 1. Yes  0. No | 1. Yes  0. No | 1. Yes  0. No |
| **R6.** Was this child given oral rehydration solution (ORS) yesterday during the day or at night? | 1. Yes  0. No | 1. Yes  0. No | 1. Yes  0. No | 1. Yes  0. No | 1. Yes  0. No | 1. Yes  0. No |

**Next I would like to ask you about some liquids that your children may have had yesterday during the day or at night. Did each child have any (ITEMS FROM LIST)?:**

|  | Name_______ | Name_______ | Name_______ | Name_______ | Name_______ | Name_______ |
| --- | --- | --- | --- | --- | --- | --- |
| **R1.** Plain water? | 1. Yes  0. No | 1. Yes  0. No | 1. Yes  0. No | 1. Yes  0. No | 1. Yes  0. No | 1. Yes  0. No |
| **R2.** Infant formula? | 1. Yes  0. No 🡪 **R4** | 1. Yes  0. No 🡪 **R4** | 1. Yes  0. No 🡪 **R4** | 1. Yes  0. No 🡪 **R4** | 1. Yes  0. No 🡪 **R4** | 1. Yes  0. No 🡪 **R4** |
| **R3.** Times yesterday (infant formula) | Times:______ | Times:______ | Times:______ | Times:______ | Times:______ | Times:______ |
| **R4.** Milk such as tinned, powdered, or fresh animal milk? | 1. Yes  0. No 🡪 **R6** | 1. Yes  0. No 🡪 **R6** | 1. Yes  0. No 🡪 **R6** | 1. Yes  0. No 🡪 **R6** | 1. Yes  0. No 🡪 **R6** | 1. Yes  0. No 🡪 **R6** |
| **R5.** Times yesterday (milk) | Times:______ | Times:______ | Times:______ | Times:______ | Times:______ | Times:______ |
| **R6.** Juice or juice drinks? | 1. Yes  0. No | 1. Yes  0. No | 1. Yes  0. No | 1. Yes  0. No | 1. Yes  0. No | 1. Yes  0. No |
| **R7.** Clear broth? | 1. Yes  0. No | 1. Yes  0. No | 1. Yes  0. No | 1. Yes  0. No | 1. Yes  0. No | 1. Yes  0. No |
| **R8.** Yogurt? | 1. Yes  0. No 🡪 **R10** | 1. Yes  0. No 🡪 **R10** | 1. Yes  0. No 🡪 **R10** | 1. Yes  0. No 🡪 **R10** | 1. Yes  0. No 🡪 **R10** | 1. Yes  0. No 🡪 **R10** |
| **R9.** Times yesterday (yogurt) | Times:______ | Times:______ | Times:______ | Times:______ | Times:______ | Times:______ |
| **R10.** Thin porridge? | 1. Yes  0. No | 1. Yes  0. No | 1. Yes  0. No | 1. Yes  0. No | 1. Yes  0. No | 1. Yes  0. No |
| **R11.** Any other liquids? | 1. Yes  0. No | 1. Yes  0. No | 1. Yes  0. No | 1. Yes  0. No | 1. Yes  0. No | 1. Yes  0. No |

**Interviewer: Please use the following script about all the foods eaten by each child yesterday.**

**"Please describe everything that (NAME) ate yesterday during the day or night, whether at home or outside the home."**

**a) Think about when (NAME) first woke up yesterday. Did (NAME) eat anything at that time?**

- **IF YES: Please tell me everything (NAME) ate at that time.**
- **PROBE: Anything else? [Until respondent says nothing else]**

**b) What did (NAME) do after that? Did (NAME) eat anything at that time?**

- **IF YES: Please tell me everything (NAME) ate at that time.**
- **PROBE: Anything else? [Until respondent says nothing else]**

**[Repeat question b above until respondent says the child went to sleep until the next day]**

**IF RESPONDENT MENTIONS MIXED DISHES LIKE A PORRIDGE, SAUCE OR STEW, PROBE:**

**c) What ingredients were in that (MIXED DISH)?**

- **PROBE: Anything else? [Until respondent says nothing else]**

**As the respondent recalls foods, mark 'yes' for the corresponding food. If foods are used in small amounts for seasoning or as a condiment, include them under the condiments food group.**

**Once the respondent finished recalling foods eaten, read each group where 'yes' was not marked and ask the following question:**

**d) Yesterday during the day or night, did (NAME) drink/eat any (food group items)?**

|  | Name_______ | Name_______ | Name_______ | Name_______ | Name_______ | Name_______ |
| --- | --- | --- | --- | --- | --- | --- |
| Porridge, bread, rice, noodles, or other foods made from grains | 1. Yes  0. No | 1. Yes  0. No | 1. Yes  0. No | 1. Yes  0. No | 1. Yes  0. No | 1. Yes  0. No |
| Pumpkin, carrots, squash, or sweet potatoes that are yellow or orange inside | 1. Yes  0. No | 1. Yes  0. No | 1. Yes  0. No | 1. Yes  0. No | 1. Yes  0. No | 1. Yes  0. No |
| White potatoes, white yams, manioc, cassava, or any other foods made from roots | 1. Yes  0. No | 1. Yes  0. No | 1. Yes  0. No | 1. Yes  0. No | 1. Yes  0. No | 1. Yes  0. No |
| Any dark green leafy vegetables | 1. Yes  0. No | 1. Yes  0. No | 1. Yes  0. No | 1. Yes  0. No | 1. Yes  0. No | 1. Yes  0. No |
| Ripe mangoes, ripe papayas | 1. Yes  0. No | 1. Yes  0. No | 1. Yes  0. No | 1. Yes  0. No | 1. Yes  0. No | 1. Yes  0. No |
| Any other fruits or vegetables | 1. Yes  0. No | 1. Yes  0. No | 1. Yes  0. No | 1. Yes  0. No | 1. Yes  0. No | 1. Yes  0. No |
| Liver, kidney, heart, or other organ meats | 1. Yes  0. No | 1. Yes  0. No | 1. Yes  0. No | 1. Yes  0. No | 1. Yes  0. No | 1. Yes  0. No |
| Any meat, such as beef, pork, lamb, goat, chicken, or duck | 1. Yes  0. No | 1. Yes  0. No | 1. Yes  0. No | 1. Yes  0. No | 1. Yes  0. No | 1. Yes  0. No |
| Eggs | 1. Yes  0. No | 1. Yes  0. No | 1. Yes  0. No | 1. Yes  0. No | 1. Yes  0. No | 1. Yes  0. No |
| Fresh or dried fish, shellfish, or seafood | 1. Yes  0. No | 1. Yes  0. No | 1. Yes  0. No | 1. Yes  0. No | 1. Yes  0. No | 1. Yes  0. No |
| Any foods made from beans, peas, lentils, nuts, or seeds | 1. Yes  0. No | 1. Yes  0. No | 1. Yes  0. No | 1. Yes  0. No | 1. Yes  0. No | 1. Yes  0. No |
| Cheese, yogurt, or other milk products | 1. Yes  0. No | 1. Yes  0. No | 1. Yes  0. No | 1. Yes  0. No | 1. Yes  0. No | 1. Yes  0. No |
| Any oil, fats, or butter, or foods made with any of these | 1. Yes  0. No | 1. Yes  0. No | 1. Yes  0. No | 1. Yes  0. No | 1. Yes  0. No | 1. Yes  0. No |
| Any sugary foods such as chocolates, sweets, candies, pastries, cakes, or biscuits | 1. Yes  0. No | 1. Yes  0. No | 1. Yes  0. No | 1. Yes  0. No | 1. Yes  0. No | 1. Yes  0. No |
| Condiments for flavor, such as chilies, spices, herbs, or fish powder | 1. Yes  0. No | 1. Yes  0. No | 1. Yes  0. No | 1. Yes  0. No | 1. Yes  0. No | 1. Yes  0. No |
| Grubs, snails, or insects | 1. Yes  0. No | 1. Yes  0. No | 1. Yes  0. No | 1. Yes  0. No | 1. Yes  0. No | 1. Yes  0. No |
| Foods made with red palm oil, red palm nut, or red palm nut pulp sauce | 1. Yes  0. No | 1. Yes  0. No | 1. Yes  0. No | 1. Yes  0. No | 1. Yes  0. No | 1. Yes  0. No |

**We are almost done with questions about your child's eating. I have just a couple more questions. [Please fill out for each child under 5 years].**

|  | Name_______ | Name_______ | Name_______ | Name_______ | Name_______ | Name_______ |
| --- | --- | --- | --- | --- | --- | --- |
| **R1.** Did this child eat any solid, semi-solid, or soft foods yesterday during the day or at night? | 1. Yes  0. No 🡪 **R3** | 1. Yes  0. No 🡪 **R3** | 1. Yes  0. No 🡪 **R3** | 1. Yes  0. No 🡪 **R3** | 1. Yes  0. No 🡪 **R3** | 1. Yes  0. No 🡪 **R3** |
| **R2.** How many times did this child eat solid, semi-solid, or soft foods other than liquids yesterday during the day or at night? | Times:______ | Times:______ | Times:______ | Times:______ | Times:______ | Times:______ |
| **R3.** Did this child drink anything from a bottle with a nipple yesterday during the day or night? | 1. Yes  0. No | 1. Yes  0. No | 1. Yes  0. No | 1. Yes  0. No | 1. Yes  0. No | 1. Yes  0. No |

**Vaccinations**

**branch_vacc** Does the respondent have a biological child under the age of 5 years?

1. No 🡪 **Skip to next section**
2. Yes

**Please fill in the information below for each child under five years of age.** For each of the vaccines, please record from the vaccine card if present. If not present, read the question to ask the respondent if the child has had the vaccine.

| Vaccine | Question | Name_______ | Name_______ | Name_______ | Name_______ | Name_______ | Name_______ |
| --- | --- | --- | --- | --- | --- | --- | --- |
|  | Do you have the Maternal Child Health Book where the child's vaccinations are written down? | 1. Yes  2. Never had book  3. Had book, but is unavailable  66. Prefers not to show book  99. Don't know | 1. Yes  2. Never had book  3. Had book, but is unavailable  66. Prefers not to show book  99. Don't know | 1. Yes  2. Never had book  3. Had book, but is unavailable  66. Prefers not to show book  99. Don't know | 1. Yes  2. Never had book  3. Had book, but is unavailable  66. Prefers not to show book  99. Don't know | 1. Yes  2. Never had book  3. Had book, but is unavailable  66. Prefers not to show book  99. Don't know | 1. Yes  2. Never had book  3. Had book, but is unavailable  66. Prefers not to show book  99. Don't know |
| BCG | A BCG vaccination against tuberculosis, that is, an injection in the arm or shoulder that usually causes a scar? | 1. Yes  0. No  99.Don’t know | 1. Yes  0. No  99.Don’t know | 1. Yes  0. No  99.Don’t know | 1. Yes  0. No  99.Don’t know | 1. Yes  0. No  99.Don’t know | 1. Yes  0. No  99.Don’t know |
| Polio 1 | Polio vaccine, that is, drops in the mouth? | 1. Yes  0. No  99.Don’t know | 1. Yes  0. No  99.Don’t know | 1. Yes  0. No  99.Don’t know | 1. Yes  0. No  99.Don’t know | 1. Yes  0. No  99.Don’t know | 1. Yes  0. No  99.Don’t know |
| Polio 2 |  | 1. Yes  0. No  99.Don’t know | 1. Yes  0. No  99.Don’t know | 1. Yes  0. No  99.Don’t know | 1. Yes  0. No  99.Don’t know | 1. Yes  0. No  99.Don’t know | 1. Yes  0. No  99.Don’t know |
| Polio 3 |  | 1. Yes  0. No  99.Don’t know | 1. Yes  0. No  99.Don’t know | 1. Yes  0. No  99.Don’t know | 1. Yes  0. No  99.Don’t know | 1. Yes  0. No  99.Don’t know | 1. Yes  0. No  99.Don’t know |
| Penta 1 | A Pentavalent vaccination, that is, an injection given in the left outer thigh, sometimes at the same time as polio drops? | 1. Yes  0. No  99.Don’t know | 1. Yes  0. No  99.Don’t know | 1. Yes  0. No  99.Don’t know | 1. Yes  0. No  99.Don’t know | 1. Yes  0. No  99.Don’t know | 1. Yes  0. No  99.Don’t know |
| Penta 2 |  | 1. Yes  0. No  99.Don’t know | 1. Yes  0. No  99.Don’t know | 1. Yes  0. No  99.Don’t know | 1. Yes  0. No  99.Don’t know | 1. Yes  0. No  99.Don’t know | 1. Yes  0. No  99.Don’t know |
| Penta 3 |  | 1. Yes  0. No  99.Don’t know | 1. Yes  0. No  99.Don’t know | 1. Yes  0. No  99.Don’t know | 1. Yes  0. No  99.Don’t know | 1. Yes  0. No  99.Don’t know | 1. Yes  0. No  99.Don’t know |
| Measles | A measles injection - that is, a shot in the arm at the age of 9 months or older - to prevent him/her from getting measles? | 1. Yes  0. No  99.Don’t know | 1. Yes  0. No  99.Don’t know | 1. Yes  0. No  99.Don’t know | 1. Yes  0. No  99.Don’t know | 1. Yes  0. No  99.Don’t know | 1. Yes  0. No  99.Don’t know |
| Vitamin A | Within the last six months, was the child given a vitamin A dose? | 1. Yes  0. No  99.Don’t know | 1. Yes  0. No  99.Don’t know | 1. Yes  0. No  99.Don’t know | 1. Yes  0. No  99.Don’t know | 1. Yes  0. No  99.Don’t know | 1. Yes  0. No  99.Don’t know |
| Pneumo 1 | A Pneumococcal vaccination, that is, an injection given in the right outer thigh, sometimes at the same time as polio drops or the Pentavalent vaccination? | 1. Yes  0. No  99.Don’t know | 1. Yes  0. No  99.Don’t know | 1. Yes  0. No  99.Don’t know | 1. Yes  0. No  99.Don’t know | 1. Yes  0. No  99.Don’t know | 1. Yes  0. No  99.Don’t know |
| Pneumo 2 |  | 1. Yes  0. No  99.Don’t know | 1. Yes  0. No  99.Don’t know | 1. Yes  0. No  99.Don’t know | 1. Yes  0. No  99.Don’t know | 1. Yes  0. No  99.Don’t know | 1. Yes  0. No  99.Don’t know |
| Pneumo 3 |  | 1. Yes  0. No  99.Don’t know | 1. Yes  0. No  99.Don’t know | 1. Yes  0. No  99.Don’t know | 1. Yes  0. No  99.Don’t know | 1. Yes  0. No  99.Don’t know | 1. Yes  0. No  99.Don’t know |

**HIV**

*Now I would like to talk about something else. As a reminder, all of your answers will be kept confidential.*

**hiv_q1** Have you ever heard of an illness called HIV/AIDS?

1. No **🡪 skip to next section**
2. Yes

**hiv_q2** I don't want to know the results, but have you ever been tested for HIV/the AIDS virus?

1. No
2. Yes 🡪 **hiv_q4**

99. Don’t know

66. Prefer not to answer 🡪 **hiv_q7**

**hiv_q3** What is keeping you from taking an HIV test? **[Select all that apply]**

**🡪 hiv_q7 after this question**

1. Denial or fear of accepting status
2. Fear of isolation or stigma
3. Religious beliefs
4. Fear of taking HIV medicine
5. Fear of spouse finding out
6. Fear of relatives or friends finding out
7. Fear of other community members finding out
8. Fear of employer finding out
9. Concerns about confidentiality
10. Cost of test and/or treatment
11. I don't know where to get an HIV test
12. Other (specify): __________________________

**hiv_q4** Where have you been tested? [Select all that apply]

1. Health facility when I was pregnant
2. Health facility when I delivered
3. Health facility when I was treated as an outpatient
4. Heath facility when I was treated as an impatient
5. Health facility when I went for the test
6. Outreach event in the community
7. At a voluntary counseling and testing enter
8. Other (specify) ____________________________

**hiv_q5** How many months ago was your most recent HIV test? _____________________

**hiv_q5b** I don't want to know the results, but did you get the results of the test?

1. No
2. Yes

**hiv_q6** Did you share the results of the test with your partner(s) or spouse(s)?0

1. Yes
2. No
3. Never received results

99. Not applicable or don’t remember

66. Prefer not to answer

*I want to assure you that I do not want to know and will not ask about the results of any HIV/AIDS tests you or your spouse or family has received. I am going to read a series of statements about HIV/AIDS. Please answer whether you strongly disagree, disagree, agree, or strongly agree with each statement.*

**hiv_q7** Some people think that those with HIV are disgusting.

1. Strongly disagree
2. Disagree
3. Agree
4. Strongly agree

**hiv_q8** Some people do not want those with HIV playing with their children.

1. Strongly disagree
2. Disagree
3. Agree
4. Strongly agree

**hiv_q9** Some people feel uncomfortable being near those with HIV.

1. Strongly disagree
2. Disagree
3. Agree
4. Strongly agree

**hiv_q10** Some people do not want to talk to others with HIV.

1. Strongly disagree
2. Disagree
3. Agree
4. Strongly agree

**hiv_q11** Some people keep distance from people with HIV.

1. Strongly disagree
2. Disagree
3. Agree
4. Strongly agree

**hiv_q12** Some people think God is punishing people with HIV.

1. Strongly disagree
2. Disagree
3. Agree
4. Strongly agree

**hiv_q13** Some people think demons are punishing people with HIV.

1. Strongly disagree
2. Disagree
3. Agree
4. Strongly agree

**hiv_q14** If a person has HIV, some community members will behave differently towards that person for the rest of his or her life.

1. Strongly disagree
2. Disagree
3. Agree
4. Strongly agree

**hiv_q15** Some people try not to touch others with HIV.

1. Strongly disagree
2. Disagree
3. Agree
4. Strongly agree

**hiv_q16** Some people are afraid of those with HIV.

1. Strongly disagree
2. Disagree
3. Agree
4. Strongly agree

**hiv_q17** Some people think that people with HIV are unclean.

1. Strongly disagree
2. Disagree
3. Agree
4. Strongly agree

**hiv_q18** Some people prefer not to have those with HIV living in their community.

1. Strongly disagree
2. Disagree
3. Agree
4. Strongly agree

**hiv_q19** Some people think that people with HIV get what they deserve.

1. Strongly disagree
2. Disagree
3. Agree
4. Strongly agree

**hiv_q20** Some people who have HIV refuse to believe that they have HIV.

1. Strongly disagree
2. Disagree
3. Agree
4. Strongly agree

**hiv_q21** Some people who have HIV feel hurt because of how others react to knowing they have HIV.

1. Strongly disagree
2. Disagree
3. Agree
4. Strongly agree

**hiv_q22** Some people with HIV feel alone.

1. Strongly disagree
2. Disagree
3. Agree
4. Strongly agree

**hiv_q23** Some people who have HIV are afraid that the people in the community will talk about them having HIV.

1. Strongly disagree
2. Disagree
3. Agree
4. Strongly agree

**hiv_q24** Some people who have HIV lose friends when they share with them that they have HIV.

1. Strongly disagree
2. Disagree
3. Agree
4. Strongly agree

**hiv_q25** Some people who have HIV lose family support when they share with them that they have HIV.

1. Strongly disagree
2. Disagree
3. Agree
4. Strongly agree

**hiv_q26** Some people who have HIV are afraid to tell their spouse or partner they have HIV.

1. Strongly disagree
2. Disagree
3. Agree
4. Strongly agree

**hiv_q27** Some people who have HIV are afraid to tell those outside their family that they have HIV.

1. Strongly disagree
2. Disagree
3. Agree
4. Strongly agree

**hiv_q28** Some people know they have HIV but are afraid to go to the clinic to get medicine for HIV.

1. Strongly disagree
2. Disagree
3. Agree
4. Strongly agree

**hiv_q29** Some people with HIV worry that others will reveal their secret.

1. Strongly disagree
2. Disagree
3. Agree
4. Strongly agree

**hiv_q30** Some people who have HIV try very hard to keep the issue of having HIV a secret.

1. Strongly disagree
2. Disagree
3. Agree
4. Strongly agree

**hiv_q31** Some people who have HIV keep their distance from others to avoid spreading the virus.

1. Strongly disagree
2. Disagree
3. Agree
4. Strongly agree

**hiv_q32** Some people who have HIV feel guilty because their family has the burden of caring for them.

1. Strongly disagree
2. Disagree
3. Agree
4. Strongly agree

**hiv_q33** Some people who have HIV will choose carefully who they tell about having HIV.

1. Strongly disagree
2. Disagree
3. Agree
4. Strongly agree

**hiv_q34** In your opinion, what do you think keeps community members from enrolling in HIV care?

**[Do not prompt] [Select all that apply]**

1. Denial or fear of accepting status
2. Fear of isolation/stigma
3. Religious beliefs
4. Fear of losing support of a spouse or being rejected by a spouse
5. Fear of violence from spouse
6. Fear of losing the support or being rejected by relatives and friends
7. Fear of other community members finding out
8. Fear of employer finding out
9. Fear of going to health facility for HIV medicine
10. Concerns about confidentiality amongst health care workers or facility staff
11. Lack of access to treatment
12. Fear of side effect
13. Lack of transportation to health facility
14. Do not think they need care
15. Other (specify) ________________________

**WASH**

**wash_q1** Currently, what is the main source for drinking water for members in your household?

**[Do not prompt]**

1. Tube well or borehole
2. Dug well - protected well
3. Dug well - unprotected well
4. Water from spring - protected spring
5. Water from spring - unprotected spring
6. Rain water
7. Tanker truck
8. Cart with small tank
9. Surface water (river/pond/stream/irrigation channel)
10. Bottled water
11. Piped water - piped into dwelling
12. Piped water - piped to yard/plot
13. Piped water - piped to neighbor
14. Piped water - public tap/standpipe
15. Other (specify) ___________________________

**wash_q2** Currently, what is the main source of water used by your household for other purposes such as cooking or washing? **[Do not prompt]**

1. Tube well or borehole
2. Dug well - protected well
3. Dug well - unprotected well
4. Water from spring - protected spring
5. Water from spring - unprotected spring
6. Rain water
7. Tanker truck
8. Cart with small tank
9. Surface water (river/pond/stream/irrigation channel)
10. Bottled water
11. Piped water - piped into dwelling
12. Piped water - piped to yard/plot
13. Piped water - piped to neighbor
14. Piped water - public tap/standpipe
15. Other (specify) ___________________________

**wash_q3** Do you usually do something to make your water safer to drink?

1. Yes
2. No **🡪 wash_q5**

99. Don’t know **🡪 wash_q5**

**wash_q4** What do you usually do to make the water safer to drink? **[Select all that apply]**

1. Boil
2. Add bleach or chlorine at home (such as Waterguard or Aquatab)
3. Use chlorine from blue dispenser at source
4. Use water filter (such as ceramic/sand/composite)
5. Solar disinfection
6. Let it stand and settle
7. Strain through a cloth
8. Cover the water container
9. Other (specify) ____________________________
10. Don't know

**wash_q5** Have you or anyone in this household, 18 years or older, received an official training in WASH, that is in water, sanitation and hygiene?

**[Explain as necessary that a training is in proper hygiene, clean water, latrine construction] [Select all that apply]**

1. Yes - 1 day training or less
2. Yes - 2 or 3 day training
3. Yes - 4 day training
4. No **🡪 wash_q7**
5. Yes - other (specify) _______________________-
6. Don't know **🡪 wash_q7**

**wash_q6** Who was responsible for the WASH training that you or other household members received? **[Select all that apply]**

1. Lwala Community Alliance
2. Another non-government or private organization
3. Government or Ministry of Health
4. Other (specify) ___________________________
5. Don’t know

**wash_q7** Has your household ever been visited by a Community Health Worker (CHW) or Umama Salama?

1. Yes
2. No **🡪 wash_q12**

99. Don’t know **🡪 wash_q12**

88. Don’t know what a CHW or Umama Salama is **🡪 wash_q12**

**wash_q8** In the past 3 months, how many times has a Community Health Worker (CHW) or Umama Salama from Lwala Community Alliance visited your household?

1. 0 visits
2. 1 visit
3. 2 visits
4. 3 visits
5. More than 3 visits
6. Don't know

**wash_q9** In the past 1 month, how many times has a Community Health Worker (CHW) or Umama Salama from Lwala Community Alliance visited your household?

1. 0 visits
2. 1 visit
3. 2 visits
4. 3 visits
5. More than 3 visits
6. Don't know

**wash_q10** What is the name of the CHW or Umama Salama who visits you most frequently?

1. Name: ________________________

99. Don’t know

**wash_q11** During the most recent visit, what did the CHW or Umama Salama from Lwala Community Alliance do when they visited your household? **[Select all that apply]**

1. Provide referral to health facility
2. Check on child's immunization status
3. Check on pregnancy status/prenatal care visit
4. Answer health questions
5. Provide family planning counseling
6. Provide emotional or social support
7. Assess health of children
8. Provide health education (such as HIV, Malaria, Diarrhea, ANC)
9. Talk about latrines or other WASH treatment
10. Talk about medications or treatment
11. Home-based diagnosis and treatment
12. Collect information about you or your family
13. Provide water purification tablets
14. Other (specify) ___________________________
15. Don't know

**wash_q12** How often would you like a CHW to visit your household in the next 1 month?

**[Explain CHW if needed]**

1. 1 time
2. 2 times
3. 3 times
4. 4 or more times
5. Never

99. Don’t know

**wash_q13** What type of services would you like to receive at your home? **[Check all that apply]**

1. None
2. Provide referral to health facility
3. Check on child's immunization status
4. Check on pregnancy status/prenatal care visit
5. Answer health questions
6. Provide family planning counseling
7. Provide emotional or social support
8. Assess health of children
9. Provide health education (such as HIV, Malaria, Diarrhea, ANC)
10. Talk about latrines or other WASH treatment
11. Talk about medications or treatment
12. Home-based diagnosis and treatment
13. Collect information about you or your family
14. Provide water purification tablets
15. Other (specify) ___________________________
16. Don't know

**Education**

**ed_q1** Teen pregnancy is an important issue in our community.

1. True
2. False

99. Don’t know

**ed_q2** As a parent, I should be involved in my child's development.

1. True
2. False

99. Don’t know

**ed_q3** Contraception should be available for teens

1. True
2. False

99. Don’t know

**ed_q4** It is okay for a girl to be married after she completes primary education

1. True
2. False

99. Don’t know

**ed_q5** A girl should get married if she becomes pregnant while in school

1. True
2. False

99. Don’t know

**branch_edu_1** Is there a child ages 5 years to 19 years living in this household?

**[Check all that apply]**

1. Yes
2. No **🡪 skip to next section**

**ed_q6** Now we are going to talk about schools and school children. Are any of your school-aged children currently not attending school, for more than the last three months?

1. No 🡪 **branch_edu_2**
2. Yes

**ed_q6b** What is the sex of the child not in school?

1. Male
2. Female
3. Both male and female children not in school

**ed_q7** What are the reasons for your child/children not being in school? **[Check all that apply]**

1. Female child is pregnant or has a baby
2. Lack of financial resources **🡪 branch_edu_2**
3. Male child impregnated someone **🡪 branch_edu_2**
4. Child got married **🡪 branch_edu_2**
5. Child got a job/employment **🡪 branch_edu_2**
6. Child refused to attend **🡪 branch_edu_2**
7. Teacher or school turned child away **🡪 branch_edu_2**
8. Other (specify) ____________________________ **🡪 branch_edu_2**

**ed_q8** Does your daughter plan to re-enroll after delivery or when the baby is older?

1. Yes
2. No - due to embarrassment or stigma
3. No - due to demands of caring for a child
4. No - she will be too old
5. No - she is not allowed to re-enroll by someone in the family
6. No - school or teacher turned her away
7. Other (specify) __________________________
8. Don't know

**branch_edu_2** Is there a male child ages 13 years to 19 years in this household?

1. No **🡪 skip to branch_edu_3**
2. Yes

**ed_q9** Have you ever talked to your SONS of adolescent age about contraceptive methods, such as condoms, IUD, injections, pills, implants?

1. Yes
2. No

99. Don’t know

**ed_q10** From the topics below, tell me those you have discussed with your SONS of adolescent age? **[Read the list] [Select all that apply]**

1. Physical changes
2. Puberty
3. Pregnancy/having babies
4. Prevention of STIs
5. Prevention of HIV/AIDS
6. Abstinence
7. Abortion
8. Use of contraceptives/family planning
9. Consequences of premarital sex
10. Violence against girls or women
11. Sexual abuse
12. None of the above

**branch_edu_3** Is there a female child ages 13 years to 19 years in this household?

1. No **🡪 skip to next section**
2. Yes

**ed_q11** Have you ever talked to your DAUGHTERS of adolescent age about contraceptive methods, such as condoms, IUD, injections, pills, implants?

1. Yes
2. No

99. Don’t know

**ed_q12** From the topics below, tell me those you have discussed with your DAUGHTERS of adolescent age? **[Read the list] [Select all that apply]**

1. Physical changes
2. Puberty
3. Pregnancy/having babies
4. Prevention of STIs
5. Prevention of HIV/AIDS
6. Abstinence
7. Abortion
8. Use of contraceptives/family planning
9. Consequences of premarital sex
10. Violence against girls or women
11. Sexual abuse
12. None of the above

**Interpersonal Violence**

**branch_ipv** Confirm: The respondent that you are speaking to is a woman, and you are in a safe and private place.

1. Yes
2. No **🡪 skip to next section**

*Now we're going to talk about what it's like to be a woman in your community. Some of these questions may be hard to answer, and we can talk about something else at any time.*

**ipv_q1** Have you ever been hit, kicked, punched, pushed, or otherwise hurt by someone in your family or in the community?

1. Yes
2. No **🡪 ipv_q2**

**ipv_q1b** When someone hurt you, did you tell your:

1. Mother
2. Father
3. Grandparent
4. Aunt/Uncle
5. Sibling
6. Teacher
7. Friend
8. Religious leader
9. Spouse / partner
10. I didn't tell anyone about this
11. Other: ______________________________

**ipv_q2** Do you feel safe in your current relationship?

1. Yes
2. No

99. Don’t know

66. Prefer not to answer

**ipv_q3** Has anyone ever forced you to have sexual activities?

1. Yes
2. No

99. Don’t know

66. Prefer not to answer

**ipv_q4** Are you afraid of your partner, or of anyone in your family or your community?

1. Yes
2. No

99. Don’t know

66. Prefer not to answer

| Does/did your (last) husband/partner ever:  **[Read list and allow for time for respondent to answer after each choice]** | | |
| --- | --- | --- |
|  | Yes | No |
| Push you, shake you, or throw something at you? |  |  |
| Slap you or twist your arm? |  |  |
| Punch you with his fist or with something that could hurt you? |  |  |
| Kick you or drag you? |  |  |
| Try to strangle you or burn you? |  |  |
| Threaten you with a knife, gun, or other type of weapon? |  |  |
| Attack you with a knife, gun, or other type of weapon? |  |  |
| Physically force you to have sexual intercourse, even when you did not want to? |  |  |
| Force you to perform other types of sexual acts when you did not want to? |  |  |
| Say or do something to humiliate you in front of other people? |  |  |
| Threaten you or someone close to you with harm? |  |  |
| Insult you or make you feel bad about yourself? |  |  |

**ipv_q7** When you were a girl, did any man in your family or household hurt a woman around you? For example, did your father ever hit or push your mother?

1. Yes
2. No

**ipv_q8** In your opinion, is a husband justified in hitting or beating his wife in the following situations: **[Check all that apply]**

1. If she goes out without telling him?
2. If she neglects the children?
3. If she argues with him?
4. If she refused to have sex with him?
5. If she burns the food?
6. I do not believe it is ever right

**ipv_q9** Which best describes how you feel about the following sentence: I feel safe in my community.

1. Strongly disagree
2. Disagree
3. Neutral
4. Agree
5. Strongly agree

**Mental Health**

| **Over the last 2 weeks, how often have you been bothered by any of the following:** | | | | |
| --- | --- | --- | --- | --- |
|  | Not at all | Several days | More than half the days | Nearly every day |
| Little interest or little happiness in doing things? |  |  |  |  |
| Feeling down, depressed, or hopeless? |  |  |  |  |
| Trouble falling or staying asleep, or sleeping too much? |  |  |  |  |
| Feeling tired or having little energy? |  |  |  |  |
| Poor appetite or overeating? |  |  |  |  |
| Feeling bad about yourself-or that you are a failure or have let yourself or your family down? |  |  |  |  |
| Trouble concentrating on things, such as reading the newspaper or watching television? |  |  |  |  |
| Moving or speaking so slowly that other people could have noticed? Or the opposite-being so fidgety or restless that you have been moving around a lot more than usual? |  |  |  |  |

**ment_q9** In general, do you think you can make decisions by yourself, freely, without consulting your spouse or family member? To which extent can you do this:

1. Never
2. Rarely
3. Sometimes
4. Always

99. Don’t know

66. Prefer not to answer

**Lwala Community Alliance (LCA) Services**

**lca_q1** Have you ever visited the Lwala Community Hospital for any clinical service for yourself, (not including taking your child or family member)? For example, antenatal care, HIV testing or care, inpatient care, outpatient care, or other treatment.

1. Yes
2. No

66. Never heard of Lwala Community Hospital **🡪 lca_q6**

99. Don’t know

**lca_q2** Has your partner/spouse ever visited the Lwala Community Hospital for any clinical service for him/herself (not including taking a child or family member to the Hospital)?

1. Yes
2. No

66. Respondent has no spouse or partner

99. Don’t know

**lca_q3** Have any children in this household received any clinical service (aside from delivery) from the Lwala Community Hospital? This includes immunizations or sickness.

1. Yes
2. No

66. No child under 18 in household

99. Don’t know

**lca_q4** Think about the last 3 months, how many times have you and members of your family visited the Lwala Community Hospital for any clinical service?

**[Add total for all members of the household]**

1. Times: ____________________

99. Don’t know

**lca_q6** Which of the following do you visit most? **[Choose from facility list]**

1. No. from facility list: ____________________

88. Other: ______________________________

**lca_q8** Why is that the facility you visit most? **[Check all that apply]**

1. Closest to me
2. Best quality
3. Relationship with staff
4. Cost/free
5. They have the drugs and supplies I need
6. They have good equipment
7. Other (specify) __________________________
8. Don't visit a facility **🡪 lca_q10**

**lca_q9** What are TWO things you would want to see improved at that facility?

**[Allow respondent to select two options only]**

1. Hours services are available
2. Confidentiality/privacy
3. Timeliness of services
4. Wait time
5. Time spent with clinician
6. Cleanliness of facility
7. Availability of drugs
8. Other (specify) ________________________

**lca_q10** Lwala Community Alliance provides a variety of services and programs. Think about yourself and current members of your household. What Lwala Community Alliance services or programs have you or someone in your household participated in within the last 1 year?

**[Select all that apply]**

1. None **🡪 lca_q13**
2. Hospital Services – Inpatient
3. Hospital Services - ANC/Delivery
4. Hospital Services - Child Welfare (Such As Immunizations)
5. Hospital Services - Other Outpatient
6. Visited By CHW Each Month
7. Farming (Nutrition) Group Participant
8. Pupil In Ereader Program (Electronic Textbooks)
9. Pupil In Health Club (After school Health Program)
10. Pupil In Girls Mentoring (In-School Mentors)
11. Girl Received Pads (Girl Student)
12. Girl Received Uniforms
13. Youth Attended Better Breaks (School Break Camp)
14. Youth Attending Youth Friendly Corner (Adolescent Health Services)
15. Youth In Out-of-school Health Club (After school)
16. Youth In Youth Peer Provider Program
17. Support Group Participant (HIV, Nutrition, Breastfeeding)
18. Table Banking Group Participant With Support From LCA/Dig (DIG Program SACCO)
19. Out Of School Girls Mentoring Participant
20. Secondary School Sponsorship
21. Kiva Loan
22. WASH Training
23. Visited By Wash Team (CLTS / Latrines)
24. Attended Community Outreach Event
25. Attended Community Dialogue Day
26. Attended WASH Tournament
27. Trained On Child Nutrition
28. Parent's Club Participant (Parent Support)
29. Attended Other Training At LCA
30. New Visions Member
31. Kangu Participant (Mama Sponsorship)
32. Village Enterprise (Microenterprise Program)

88. Other (specify) _________________________________

99. Yes, but don’t know what

**lca_q11** Of the programs and services you or your family has participated in, which has the most value for you? **[Select only one]**

1. None
2. Hospital Services – Inpatient
3. Hospital Services - ANC/Delivery
4. Hospital Services - Child Welfare (Such As Immunizations)
5. Hospital Services - Other Outpatient
6. Visited By CHW Each Month
7. Farming (Nutrition) Group Participant
8. Pupil In Ereader Program (Electronic Textbooks)
9. Pupil In Health Club (After school Health Program)
10. Pupil In Girls Mentoring (In-School Mentors)
11. Girl Received Pads (Girl Student)
12. Girl Received Uniforms
13. Youth Attended Better Breaks (School Break Camp)
14. Youth Attending Youth Friendly Corner (Adolescent Health Services)
15. Youth In Out-of-school Health Club (After school)
16. Youth In Youth Peer Provider Program
17. Support Group Participant (HIV, Nutrition, Breastfeeding)
18. Table Banking Group Participant With Support From LCA/Dig (DIG Program SACCO)
19. Out Of School Girls Mentoring Participant
20. Secondary School Sponsorship
21. Kiva Loan
22. WASH Training
23. Visited By Wash Team (CLTS / Latrines)
24. Attended Community Outreach Event
25. Attended Community Dialogue Day
26. Attended WASH Tournament
27. Trained On Child Nutrition
28. Parent's Club Participant (Parent Support)
29. Attended Other Training At LCA
30. New Visions Member
31. Kangu Participant (Mama Sponsorship)
32. Village Enterprise (Microenterprise Program)

88. Other (specify) _________________________________

**lca_q12** Have you ever recommended a Lwala Community Alliance service or program to a friend or neighbor? **[If unsure, ask if they've ever told a friend or neighbor to go to Lwala for assistance]**

1. Yes
2. No

99. Don’t know

**lca_q13** If you could, what program or service would you most like to get involved in at Lwala Community Alliance next? **[Select only one]**

1. None
2. Hospital Services – Inpatient
3. Hospital Services - ANC/Delivery
4. Hospital Services - Child Welfare (Such As Immunizations)
5. Hospital Services - Other Outpatient
6. Visited By CHW Each Month
7. Farming (Nutrition) Group Participant
8. Pupil In Ereader Program (Electronic Textbooks)
9. Pupil In Health Club (After school Health Program)
10. Pupil In Girls Mentoring (In-School Mentors)
11. Girl Received Pads (Girl Student)
12. Girl Received Uniforms
13. Youth Attended Better Breaks (School Break Camp)
14. Youth Attending Youth Friendly Corner (Adolescent Health Services)
15. Youth In Out-of-school Health Club (After school)
16. Youth In Youth Peer Provider Program
17. Support Group Participant (HIV, Nutrition, Breastfeeding)
18. Table Banking Group Participant With Support From LCA/Dig (DIG Program SACCO)
19. Out Of School Girls Mentoring Participant
20. Secondary School Sponsorship
21. Kiva Loan
22. WASH Training
23. Visited By Wash Team (CLTS / Latrines)
24. Attended Community Outreach Event
25. Attended Community Dialogue Day
26. Attended WASH Tournament
27. Trained On Child Nutrition
28. Parent's Club Participant (Parent Support)
29. Attended Other Training At LCA
30. New Visions Member
31. Kangu Participant (Mama Sponsorship)
32. Village Enterprise (Microenterprise Program)

88. Other (specify) _________________________________

**lca_q14** What are your three services or programs that you wish you had in your community?

1. ____________________________________________
2. ____________________________________________
3. ____________________________________________

**COVID-19**

**cov_q1** Have you heard of COVID-19?

1. No **🡪 skip to next section**
2. Yes

**cov_q2** Please assess your private financial situation since COVID began.

1. Improved
2. Remains the same
3. Worse
4. Don’t know

**cov_q3** Where did you hear of COVID-19? **[check all that apply]**

1. Friends or family
2. Television
3. Newspapers
4. Health workers
5. Community Health Workers that visit your home
6. Social media
7. Radio
8. Ministry of Health
9. Institute of Public Health/Center for Disease Control

**cov_q4** Have you had the following in the last two weeks? **[check all that apply]**

1. Cough
2. Congestion or runny nose
3. Shortness of breath or difficulty breathing
4. Fever of chills
5. Muscle or body aches
6. Vomiting
7. Diarrhea
8. Loss of taste
9. Loss of smell
10. None of the above

**cov_q5** Has anyone in the household had the following in the last two weeks? **[check all that apply]**

1. Cough
2. Congestion or runny nose
3. Shortness of breath or difficulty breathing
4. Fever of chills
5. Muscle or body aches
6. Vomiting
7. Diarrhea
8. Loss of taste
9. Loss of smell
10. None of the above

**cov_q6** To your knowledge, are you, or have you been, infected with COVID-19?

1. No **🡪 cov_q9**
2. Yes

**cov_q7** Was your COVID-19 infection mild or severe?

1. Mild
2. Severe

**cov_q8** Was your COVID-19 infection confirmed by a test?

1. Confirmed by a test
2. Not confirmed by a test

**cov_q9** Do you know people in your immediate social environment who are or have been infected with COVID-19 (suspected or confirmed)?

1. No **🡪 skip to table below**
2. Yes

**cov_q10** Do you know someone who died from COVID-19?

1. No
2. Yes

| **Now I would like to ask you some questions about how easy it is to access information for COVID-19. How easy or difficult would you say it is to...:** | | | | |
| --- | --- | --- | --- | --- |
|  | Very Difficult | Difficult | Easy | Very Easy |
| ...find the information you need related to COVID-19? |  |  |  |  |
| ...understand information about what to do if you think you have COVID-19? |  |  |  |  |
| ...judge if the information about COVID-19 in the media is reliable? |  |  |  |  |
| ...understand restrictions and recommendations of authorities regarding COVID-19? |  |  |  |  |
| ...follow the recommendations on how to protect yourself from COVID-19? |  |  |  |  |
| ...understand recommendations about when to stay at home from work/school, and when not to? |  |  |  |  |
| ...follow recommendations about when to stay at home from work/school, and when not to? |  |  |  |  |
| ...understand recommendations about when to engage in social activities, and when not to? |  |  |  |  |
| ...follow recommendations about when to engage in social activities, and when not to? |  |  |  |  |

**cov_q21** What do you consider to be your own probability of getting infected with COVID -19?

1. Extremely unlikely
2. Unlikely
3. Neither likely nor unlikely
4. Likely
5. Extremely likely

**cov_q22** How susceptible do you consider yourself to an infection with COVID-19?

1. Not at all susceptible
2. Somewhat susceptible
3. Neutral
4. Susceptible
5. Very susceptible

**cov_q23** How severe would contracting COVID-19 be for you (how seriously ill do you think you will be)?

1. Not severe
2. Somewhat severe
3. Moderately severe
4. Severe
5. Very severe

**cov_q24** I know how to protect myself from coronavirus

1. Not at all
2. Somewhat
3. Average
4. More than average
5. Very much so

**cov_q25** For me avoiding an infection with COVID-19 in the current situation is...

1. Extremely difficult
2. Difficult
3. Neither difficult nor easy
4. Easy
5. Extremely easy

| **During the last 7 days, which of the following measures have you taken to prevent infection from COVID-19?** | | |
| --- | --- | --- |
|  | No | Yes |
| Frequently washed my hands with soap and water for at least 20 seconds |  |  |
| Avoided touching my eyes, nose, and mouth with unwashed hands |  |  |
| Used sanitizer to clean hands when soap and water were not available |  |  |
| Avoided a social event I wanted to attend |  |  |
| Stayed at home from work because it was closed |  |  |
| Stayed at home from work by choice |  |  |
| Stayed at home from school because it was closed |  |  |
| Stayed at home from school by choice |  |  |
| Used antibiotics to prevent or treat COVID-19 |  |  |
| Wore a mask in public |  |  |
| Ensured physical distancing in public |  |  |
| Disinfected surfaces |  |  |

**cov_q38** Do you own a face mask or covering?

1. No **🡪 skip to table below**
2. Yes

**cov_q39** Does everyone living in this household have their own face mask or covering?

1. No, only one person has a face mask
2. No, but more than one person has a face mask
3. Yes

| **How much do you trust information about COVID-19 from the following sources?** | | | | | |
| --- | --- | --- | --- | --- | --- |
|  | Very little trust | Little trust | Moderate trust | Much trust | A great deal of trust |
| Television |  |  |  |  |  |
| Newspapers |  |  |  |  |  |
| Health workers |  |  |  |  |  |
| Community health workers that visit your home |  |  |  |  |  |
| Social media |  |  |  |  |  |
| Radio |  |  |  |  |  |
| Ministry of Health |  |  |  |  |  |
| Institute of Public Health/Center for Disease Control |  |  |  |  |  |
| Celebrities or social media influencers |  |  |  |  |  |
| World Health Organization (WHO) |  |  |  |  |  |
| COVID-19 Hotline |  |  |  |  |  |
| National COVID-19 information website |  |  |  |  |  |

| **How often do you use the following sources for information about COVID-19?** | | | | | |
| --- | --- | --- | --- | --- | --- |
|  | Never | Rarely | Sometimes | Often | Very Often |
| Television |  |  |  |  |  |
| Newspapers |  |  |  |  |  |
| Health workers |  |  |  |  |  |
| Community health workers that visit your home |  |  |  |  |  |
| Social media |  |  |  |  |  |
| Radio |  |  |  |  |  |
| Ministry of Health |  |  |  |  |  |
| Institute of Public Health/Center for Disease Control |  |  |  |  |  |
| Celebrities or social media influencers |  |  |  |  |  |
| World Health Organization (WHO) |  |  |  |  |  |
| COVID-19 Hotline |  |  |  |  |  |
| National COVID-19 information website |  |  |  |  |  |

**cov_q64** How often do you seek information about COVID-19?

1. Never
2. Rarely
3. Every week
4. Every day
5. Several times a day

| **How much confidence do you have that the following can handle the COVID-19 challenge well?** | | | | | | |
| --- | --- | --- | --- | --- | --- | --- |
|  | Very low confidence | Low confidence | Neutral | High confidence | Very high confidence | Not applicable |
| Your healthcare provider |  |  |  |  |  |  |
| Your employer |  |  |  |  |  |  |
| Hospitals |  |  |  |  |  |  |
| Ministry of Health |  |  |  |  |  |  |
| Institute of Public Health/Center for Disease Control |  |  |  |  |  |  |
| Schools |  |  |  |  |  |  |
| Public transportation companies |  |  |  |  |  |  |
| Police |  |  |  |  |  |  |
| Your church/place of worship |  |  |  |  |  |  |
| Lwala Community Alliance |  |  |  |  |  |  |

| **Please consider the decisions that are made in your country to reduce spread of COVID -19. I think that.... [Answers in percent certainty the statement is true]** | | | | | | | | | | | |
| --- | --- | --- | --- | --- | --- | --- | --- | --- | --- | --- | --- |
|  | 0% | 10% | 20% | 30% | 40% | 50% | 60% | 70% | 80% | 90% | 100% |
| ... many very important things happen in the world, which the public is never informed about |  |  |  |  |  |  |  |  |  |  |  |
| ... politicians usually do not tell us the true motives for their decisions |  |  |  |  |  |  |  |  |  |  |  |
| ... government agencies closely monitor all citizens |  |  |  |  |  |  |  |  |  |  |  |
| ... events which superficially seem to lack connection are often the result of secret activities |  |  |  |  |  |  |  |  |  |  |  |
| ...there are secret organizations that greatly influence political decisions |  |  |  |  |  |  |  |  |  |  |  |
| ... COVID-19 is a way for the government to make money |  |  |  |  |  |  |  |  |  |  |  |
| ... COVID-19 is a punishment from God |  |  |  |  |  |  |  |  |  |  |  |
| ... the virus that causes COVID-19 was probably created in a laboratory |  |  |  |  |  |  |  |  |  |  |  |
| ... the number of people reported as dying from coronavirus is being deliberately exaggerated by the government |  |  |  |  |  |  |  |  |  |  |  |

**cov_q84** Do you think the COVID-19 pandemic is part of a global conspiracy?

1. No
2. Yes
3. Maybe

**cov_q85** Have you been tested for COVID-19

1. No **🡪 cov_q87**
2. Yes

**cov_q86** Why were you tested? **[check all that apply]**

1. I was exposed to someone with COVID-19
2. I had symptoms concerning for COVID-19
3. I wanted to know if I had COVID-19
4. Other

**cov_q87** If you have symptoms of COVID-19 - will you get tested if you have the opportunity?

1. I would get tested for sure
2. I may not get tested **🡪 cov_q89**

**cov_q88** I would get tested for sure because... **🡪 skip to next table after this question**

1. I want to receive the appropriate care in case of a positive test
2. This is my responsibility as a citizen
3. I would face penalties if I did not
4. I believe this helps stop the spread of COVID-19
5. This way I can protect other people
6. My friends and family would expect me to get tested

**cov_q89** I may not get tested because...

1. Getting tested would cost money (e .g. transportation, buying the test, taking time off work)
2. I do not know where to go to be tested
3. It is too time-consuming to get teste
4. This will result in loss of income for me due to quarantine while waiting to get the results
5. This would result in loss of income for me if I get a positive test
6. People might blame me for my actions if I get a positive test
7. I might face fines or other penalties if I had violated official COVID restrictions
8. I do not trust authorities with my personal data
9. I do not believe COVID-19 exists
10. There is nothing I can do, even if I get a positive test
11. I am not able to self-isolate in case I get a positive test
12. I do not think the tests are reliable
13. I am worried people will treat me badly i f I get a positive tesr
14. I am worried I will get infected at the testing site
15. I think testing will be painful

| **Please indicate, to which degree you support the following decisions:** | | | | | |
| --- | --- | --- | --- | --- | --- |
|  | Strongly disagree | Disagree | Neither agree nor disagree | Agree | Strongly agree |
| Compulsory face masks in closed public spaces |  |  |  |  |  |
| Restricting the number of people that can gather together |  |  |  |  |  |
| Closing schools |  |  |  |  |  |
| Restricting how close people can sit together on busses and other public transportation |  |  |  |  |  |
| Restricting how late people can stay out (curfew) |  |  |  |  |  |

| **Within the last 2 weeks, have you done the following...?** | | |
| --- | --- | --- |
|  | No | Yes |
| Avoided people that I thought might infect me, based on their tribe/region |  |  |
| Drank more alcohol than I did before the pandemic |  |  |
| Ate less food than I did before the pandemic |  |  |
| Smoked more than I did before the pandemic |  |  |
| Postponed vaccination for myself or my child due to COVID-19 (non-COVID vaccine) |  |  |
| Avoided going to the doctor for a non-COVID-19-related problem |  |  |
| Bought drugs that I heard are good for treating COVID-19 |  |  |

*COVID-19 vaccines are currently available. I would now like to ask you questions about these vaccines.*

**cov_q102** When a COVID-19 vaccine is available to you, will you get it?

1. No
2. Yes

**cov_q116** Apart from COVID-19, do you think everyone should be vaccinated according to the national vaccination schedule?

1. No
2. Yes

**cov_q103** I believe a vaccine can help control the spread of COVID-19

1. Strongly disagree
2. Disagree
3. Neither agree nor disagree
4. Agree
5. Strongly agree

**cov_q104** If I knew I had been infected with COVID-19 before, I would not get the vaccine even if it were available

1. Strongly disagree
2. Disagree
3. Neither agree nor disagree
4. Agree
5. Strongly agree

**cov_q105** When everyone else is vaccinated against COVID-19, then I don't have to get vaccinated

1. Strongly disagree
2. Disagree
3. Neither agree nor disagree
4. Agree
5. Strongly agree

| **My decision of whether or not to get vaccinated depends on:** | | | | | |
| --- | --- | --- | --- | --- | --- |
|  | Not at all | Slightly | Somewhat | Moderately | Extremely |
| Country in which the vaccine is produced |  |  |  |  |  |
| Recommendation from my family doctor/healthcare provider |  |  |  |  |  |
| Recommendation from a Community Health Worker that visits my home |  |  |  |  |  |
| Recommendation of the Ministry of Health |  |  |  |  |  |
| Whether the vaccine has been in use for a long time with no serious side-effects |  |  |  |  |  |
| Whether the vaccine is used in other countries |  |  |  |  |  |
| Risk of getting infected with COVID-19 at the time when the vaccine is available |  |  |  |  |  |
| How easy it is to get the vaccine |  |  |  |  |  |
| Whether the vaccine is free of charge |  |  |  |  |  |
| Whether a high vaccination uptake would lift restrictions on movement and gathering in groups |  |  |  |  |  |

| **I am going to read a series of statements about COVID-19. Please answer whether you strongly disagree, disagree, agree, or strongly agree with each statement.** | | | | |
| --- | --- | --- | --- | --- |
|  | Strongly disagree | Disagree | Agree | Strongly agree |
| Some people think that those with who have had COVID-19 are disgusting |  |  |  |  |
| Some people do not want those who have had COVID-19 playing with their children |  |  |  |  |
| Some people feel uncomfortable being near those who have had COVID-19 |  |  |  |  |
| Some people do not want to talk to others who have had COVID-19 |  |  |  |  |
| Some people keep distance from people who have had COVID-19 |  |  |  |  |
| If a person has had COVID-19, some community members will behave differently towards that person for the rest of his or her life |  |  |  |  |
| Some people try not to touch others who have had COVID-19 |  |  |  |  |
| Some people are afraid of those who have had COVID-19 |  |  |  |  |
| Some people think that people who have had COVID-19 are unclean |  |  |  |  |
| Some people prefer not to have those who have had COVID-19 living in their community |  |  |  |  |
| Some people think that people who have had COVID-19 get what they deserve |  |  |  |  |
| Some people who have had COVID-19 feel hurt because of how others react to knowing they have had COVID-19 |  |  |  |  |
| Some people who have had COVID-19 feel alone |  |  |  |  |
| Some people who have had COVID-19 are afraid that other people in the community will talk about them having had COVID-19 |  |  |  |  |
| Some people who have had COVID-19 lose friends when they share with them they have had COVID-19 |  |  |  |  |
| Some people who have had COVID-19 are afraid to tell those outside their family that they have had COVID-19 |  |  |  |  |
| Some people who have had COVID-19 worry that others will reveal their secret |  |  |  |  |
| Some people who have had COVID-19 try very hard to keep the issue of having had COVID-19 a secret |  |  |  |  |
| Some people who have had COVID-19 keep their distance from others to avoid spreading the COVID-19 virus |  |  |  |  |
| Some people who have had COVID-19 feel guilty because their family has the burden of caring for them |  |  |  |  |
| Some people who have had COVID-19 will choose carefully who they tell about having had COVID-19 |  |  |  |  |

**Observational Information**

**obv_q1** Did you sleep under a mosquito net last night?

1. Yes
2. No 🡪 **obv_q3**

99. Don’t know 🡪 **obv_q3**

**obv_q2** Can you show me the mosquito net you slept under?

1. Yes - good quality net observed
2. Yes - poor quality net observed
3. Yes - no net observed
4. Other (specify)_____________________-
5. No permission to see

**obv_q3** Did the youngest child in the household sleep under a mosquito net last night?

1. Yes
2. No 🡪 **obv_q5**

99. Don’t know 🡪 **obv_q5**

66. No child in the household 🡪 **obv_q5**

**obv_q4** Can you show me the mosquito net that youngest child slept under?

1. Yes - same net as respondent (good quality)
2. Yes - same net as respondent (poor quality)
3. Yes, different net as respondent (good quality)
4. Yes - different net as respondent (poor quality)
5. Yes - no net observed
6. Other (specify) _________________________
7. No permission to see

**obv_q5** How many people slept in this household last night? _____________________

**obv_q5b** How many of these people slept under a mosquito net? _____________________

**obv_q6** How many mosquito nets does your family have in this house? __________________

**[if 0, then skip to obv_q12]**

**obv_q7** How many months ago did you get your newest mosquito net? __________________

**obv_q8** How many of the nets in your household were donated?

1. Number of nets: _________________
2. None

99. Don’t know

**obv_q9** How many of the nets in your household were purchased?

1. Number of nets: _________________
2. None

99. Don’t know

**obv_q10** How many of your mosquito nets need to be treated with insecticide? ___________

**obv_q11** Which one needs to be soaked?

1. None
2. A donated net
3. A purchased net
4. Both

99. Don’t know

**obv_q12** Is there a season of the year when you do NOT need to use mosquito nets?

**[Select all that apply]**

1. No
2. Yes – dry season
3. Yes – short rains
4. Yes – long rains

99. Don’t know

**obv_q13** How many habitable rooms does this household occupy in its main dwelling (do not count storerooms, toilets, bathrooms)? _____________________

**obv_q26** How many rooms in this household are used for sleeping? _____________________

**obv_q14** Main material of the floor (record observation):

1. Earth/Sand
2. Dung
3. Wood Planks
4. Palm/Bamboo
5. Parquet or Polished Wood
6. Vinyl or Asphalt Strips
7. Ceramic Tiles
8. Cement
9. Carpet
10. Other ________________________

**obv_q15** What is the main source of lighting fuel for the household?

1. Collected firewood/purchased firewood/grass or dry cells (torch)
2. Paraffin/Candles/Biogas
3. Electricity
4. Solar or gas
5. Other (specify)__________________________
6. Prefer not to answer
7. Don't know

**branch_garden** Does respondent have a kitchen garden?

1. No 🡪 **obv_q18**
2. Yes

**obv_q16** Can you show me your kitchen garden? **[Measure the kitchen garden in steps]**

1. Steps: ______________________

66. No permission to see

**obv_q17** Mark whether the garden has enriched, raised beds:

1. No
2. Yes

**[For OBV_Q18 - OBV_Q20, observe presence of water and soap at the place for hand washing and record observation]**

**obv_q18** Can you please show me where members of your household most often wash their hands?

1. Observed - fixed place
2. Observed – mobile
3. Not observed/Not in dwelling or yard **🡪 obv_q21**
4. Other (specify) ______________________ **🡪 obv_q21**
5. No permission to see **🡪 obv_q21**

**obv_q19** Mark if water is available

1. No
2. Yes

**obv_q20** Mark if soap is available

1. No
2. Yes

**obv_q21** Can you now show me where you put your dishes after washing them?

**[Mark where dishes dry]**

1. Outside drying rack
2. Outside on ground
3. Inside on rack or table
4. Inside on ground
5. In a bucket or basin
6. Not observed
7. Other (specify) ______________________
8. Don't know
9. No permission to see

**obv_q22** I'd like to take a look at where you cook - can you show me that place?

**[Mark where cooking takes place]**

1. Outdoors
2. Separate kitchen – ventilated
3. Separate kitchen - not ventilated
4. In the home – ventilated
5. In the home - not ventilated
6. No permission to see
7. Other (specify) ______________________

**obv_q23** What fuel do you normally use when you cook?

1. Firewood - open stove
2. Firewood - improved stove
3. Charcoal/Jiko
4. Paraffin
5. Gas stove
6. Electric stove
7. Other (specify) ______________________
8. Don't know

**obv_q24** Currently, what kind of toilet facility do you and members of your household use?

1. Flush to piped sewer system
2. Flush to septic tank
3. Flush to pit latrine
4. Flush to somewhere else
5. Flush, don't know where
6. Ventilated improved pit latrine
7. Pit latrine with slab
8. Pit latrine without slab/Open pit
9. Composting toilet
10. Bucket toilet
11. Hanging toilet/Hanging latrine
12. No facility/bush/field **🡪 int_end**
13. Other (specify) ______________________

**obv_q24_share** Do you share this toilet facility with other households?

1. No
2. Yes

**obv_q25** Can you show me the latrine? **[Record condition of latrine]**

1. Not observed
2. Good condition
3. Poor condition but useable
4. Not usable/closed up
5. Other (specify) ______________________
6. No permission to see

**int_end** Interview date and end time: ______________________

**Facility List**

| 0 | Adoma Medical Center | 44 | Zidkijah Medical Center |
| --- | --- | --- | --- |
| 1 | Awendo Jiwdendi Clinic | 45 | Kamagambo SDA Dispensary |
| 2 | Awendo Sub County Hospital | 46 | Kangeso Dispensary |
| 3 | Granny Medical Clinic | 47 | Kochola Dispensary |
| 4 | Ombo Bita Dispensary | 48 | Ndege Oriedo Dispensary |
| 5 | Otacho Dispensary | 49 | Ngere Health Centre |
| 6 | Rapcom Nursing and Maternity Home | 50 | Ngodhe Dispensary |
| 7 | Sony Medical Centre | 51 | Relyne Medical Centre |
| 8 | Acela Community Clinic | 52 | Verna Health Centre |
| 9 | Angogo dispensary | 53 | Lwala Community Hospital |
| 10 | Jevros Clinic | 54 | Minyenya Health Centre |
| 11 | Kuja Dispensary | 55 | Hoplan Medical Center |
| 12 | Kwoyo Kodalo Dispensary | 56 | Kitere Dispensary |
| 13 | Obama dispensary | 57 | Ongo Health Centre |
| 14 | Ranen (SDA) Dispensary | 58 | Rongo University Medical Center |
| 15 | Ranen Ochuna Medical Centre | 59 | Neoglobal Medicare Hospital |
| 16 | Alliance community medical centre | 60 | Ombo Kowiti Dispensary |
| 17 | Angaga Dispensary | 61 | Omulo Dispensary |
| 18 | Bonde Dispensary (Awendo) | 62 | Piny Owacho Dispensary |
| 19 | Dalcoo medical centre | 63 | St. Paul's Dawa Medical Clinic |
| 20 | Liza Medical Clinic | 64 | Uriri Sub County Hospital |
| 21 | Mariwa Health Centre | 65 | Oyani Sub County Hospital |
| 22 | Ng'ong'a Dispensary | 66 | St Askad Medical clinic |
| 23 | Oasis Care Centre Andapol Clinic Awendo | 67 | Thim Jope Dispensary |
| 24 | P&D Health Clinic Ulanda | 68 | Koloo Dispensary |
| 25 | Sare family medical centre | 69 | Nyamasare Dispensary |
| 26 | Tuungane Center (Awendo) | 70 | Ongito Dispensary |
| 27 | Ulanda Dispensary | 71 | Othoro Sub County Hospital |
| 28 | Dede Health Centre | 72 | Rae Kondiala Dispensary |
| 29 | Rabondo Dispensary | 73 | St Monica Rapogi Hospital |
| 30 | Siruti Dispensary | 74 | Wangiya dispensary |
| 31 | Jamii Dental Clinic- Rongo | 75 | Bware Health Center |
| 32 | Kogelo Oasis Medical Clinic | 76 | Kamsaki Dispensary |
| 33 | Ladopharma Medical Centre-Annex Rongo | 77 | Kolwal Dispensary |
| 34 | Ladopharma Nursing Home Ltd (Rongo) | 78 | Lwala Nyarago Dispensary |
| 35 | Lenmek Medical Clinic | 79 | Neocare Memorial Hospital |
| 36 | Pona VCT | 80 | Nyasoko Dispensary |
| 37 | Riosiri Centre Medical Clinic-Rongo | 81 | Koringo Dispensary |
| 38 | Rongo Bahati Medical Centre | 82 | LELA Dispensary |
| 39 | Rongo Sub County Hospital | 83 | Midida Dispensary |
| 40 | Rosewood Nursing Home | 84 | Nyamage Dispensary |
| 41 | Royal Medical Hospital and Maternity Home Limited | 85 | Osogo Dispensary |
| 42 | Tudor Healthcare Rongo | 86 | Oyani (SDA) Dispensary |
| 43 | Tuungane Centre Rongo | 87 | Sibuoche Dispensary |
